# Review of ZnO Binary and Ternary Composite Anodes for Lithium-Ion Batteries

**DOI:** 10.3390/nano11082001

**Published:** 2021-08-04

**Authors:** Vu Khac Hoang Bui, Tuyet Nhung Pham, Jaehyun Hur, Young-Chul Lee

**Affiliations:** 1 Department of BioNano Technology, Gachon University, Seongnam-si 13120, Korea; hoangvu2101@gachon.ac.kr; 2 Phenikaa University Nano Institute (PHENA), PHENIKAA University, Hanoi 12116, Vietnam; nhungpham240694@gmail.com; 3 Department of Chemical and Biological Engineering, Gachon University, Seongnam-si 13120, Korea

**Keywords:** ZnO, composites, binary, ternary, LIBs, anode

## Abstract

To enhance the performance of lithium-ion batteries, zinc oxide (ZnO) has generated interest as an anode candidate owing to its high theoretical capacity. However, because of its limitations such as its slow chemical reaction kinetics, intense capacity fading on potential cycling, and low rate capability, composite anodes of ZnO and other materials are manufactured. In this study, we introduce binary and ternary composites of ZnO with other metal oxides (MOs) and carbon-based materials. Most ZnO-based composite anodes exhibit a higher specific capacity, rate performance, and cycling stability than a single ZnO anode. The synergistic effects between ZnO and the other MOs or carbon-based materials can explain the superior electrochemical characteristics of these ZnO-based composites. This review also discusses some of their current limitations.

## 1. Introduction

**Figure 1:**
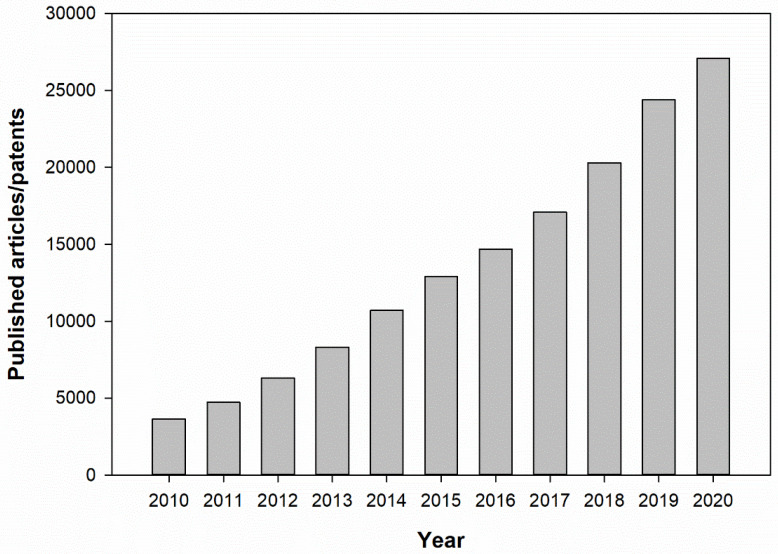
Published articles/patents related to MOs and their composites for LIB applications. Data collected from Google Scholar (https://scholar.google.com) (accessed on 15 June 2021) database with keyword: “Metal oxide lithium-ion batteries”.

Rechargeable lithium-ion batteries (LIBs) are widely used owing to their high specific energy, high electrochemical performance, and extended lifetime [1]. They are extensively adopted to power various electronic appliances, such as laptops and mobile phones. However, graphite, which is used as an anode in LIBs, limits their practical applications because of its low theoretical capacity (372 mAh g^−1^) [2]. Semiconductor metal oxides (MOs) have the potential to enhance the performance of LIBs because of their higher theoretical capacity and safety than the traditional materials, such as carbon materials [3]. In the past ten years, the literature and patents on MOs and their composites for LIB applications have drastically increased (Figure 1), which is a trend that is expected to continue. Among MOs, zinc oxide (ZnO) (978 mAh g^−1^) has an excellent theoretical capacity (Table 1), which is only slightly lower than that of ferric oxide (Fe_2_O_3_). Compared to other MOs, ZnO not only has a higher theoretical capacity but also a lower cost, ease of synthesis, various synthesis methods, chemical stability, and different morphologies [4]. Therefore, in this study, ZnO-based composites were selected as the target.

As mentioned above, ZnO is a promising anode material candidate for LIBs owing to its high theoretical capacity (978 mAh g^−1^) [5]. ZnO is a low-band gap semiconductor (3.37 eV) with unique properties such as a high exciton binding energy (60 meV), photoelectric response, and electron mobility. The above features along with its good thermal and chemical stability render ZnO useful for various applications [6]. The general electrochemical mechanism for a ZnO anode in an LIB is as follows [7]:

ZnO + 2Li^+^ + 2e^−^ ↔ Zn + Li_2_O (1)

Zn + Li^+^ + e^−^ ↔ LiZn (2)

Equation (1) is a conversion reaction, and (2) is an alloying–dealloying reaction. In (1), ZnO captures more lithium ions (Li^+^) than traditional anodes, which proves beneficial during (2) [7]. Materials that undergo both conversion and alloying–dealloying reactions have higher capacities than those that involve only alloying reactions [8]. Unlike tin oxide (SnO_2_), which undergoes irreversible conversion and reversible alloying–dealloying reactions [9], for ZnO, both reactions are reversible [7,10].

However, ZnO has numerous limitations such as its slow chemical reaction kinetics, intense capacity fading on potential cycling, and low rate capability [20]. Moreover, it tends to aggregate and undergo a remarkable volume change (228%) during the charge/discharge cycles [7,21]. Although a thin layer formation occurs during the first cycle, it is extremely thin to be robust to the volume variation in ZnO. Thus, nanocracks can form inside ZnO and lead to continuous growth of the solid electrolyte interface (SEI) layer [22,23]. To realize LIB anodes with high reversible capacity, structural stability, and activity, materials with high conductivity are necessary [5]. 

Some strategies to increase the electrochemical performance of ZnO are to realize nanoscale-sized particles and to fabricate different nanoarchitectures, such as nanospheres, nanorods, nanotubes, and nanosheets [24,25,26,27]. Nanostructured MOs can decrease the Li^+^ diffusion time and enhance the rate performance [9]. ZnO nanoarchitectures not only provide a barrier against the volume variation during the cycling process but also enhance the electrode/electrolyte contact area [28]. However, such different nanosized and morphological ZnO forms still have a low intrinsic electrical conductivity and exhibit a large volume change during the charge/discharge process [29]. Concurrently, composites of ZnO and other materials can be formed to increase its initial capacity and decrease the degradation during the charge/discharge process [7]. In addition, coating ZnO on materials such as carbon can alleviate the volume change problem [7,30]. Thus, ZnO-based nanomaterials offer a high electrochemical conductivity, a short transport path for Li^+^, and an extended lifetime [7]. In this review, we focus on ZnO-based binary and ternary composites.

## 2. ZnO Binary Composites

The alloying reaction of MO anodes in LIBs can cause a large volume change and structural stress in the anodes, thus damaging them, such as through cracking and pulverization [31]. These problems not only lower the LIB performance but also threaten safety. One strategy to overcome these issues is to design composite anodes of carbon materials and MOs [20].

### 2.1. ZnO–MO Composites

**Figure 2:**
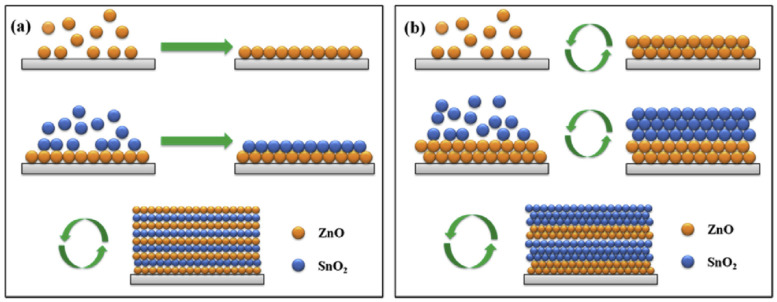
Synthesis of intermixed (**a**) and nanolaminated (**b**) ZnO–SnO_2_ composite films by ALD. Reprinted with permission from [6]. Copyright 2019, Elsevier.

In the binary MO composites, typically, Li^+^ insertion/extraction occurs for both transition Mos during the charge/discharge process, which enhances the kinetics of the reduction–oxidation reactions and improves the electrical conductivity compared to the individual oxides; these, in turn, enhance the anodic performance in battery applications [32]. ZnO–SnO_2_ composite anodes have been synthesized using various methods including hydrothermal methods, ball milling, layer-by-layer approaches, chemical vapor deposition, and physical vapor deposition [33,34,35,36,37]. Recently, Zhao et al. (2019) prepared two types of ZnO–SnO_2_ composites by atomic layer deposition (ALD): (1) intermixed films in which Zn, Sn, and O were atomically mixed in a single amorphous layer, and (2) nanolaminated films with a well-defined interfacial formation between the ZnO and SnO_2_ layers (Figure 2). The ALD processes were 100 × (2 ZnO + 3 SnO_2_) and 100 × (20 ZnO + 30 SnO_2_) for the intermixed (Figure 2a) and nanolaminated (Figure 2b) ZnO–SnO_2_ composites, respectively. X-ray photoelectron spectroscopy (XPS) data confirmed the co-existence of ZnO and SnO_2_ in the as-prepared composites. The Zn, Sn, and O atomic concentrations remained unchanged over the entire intermixed ZnO–SnO_2_ thin film, which indicated uniform ZnO and SnO_2_ mixing. In contrast, the Zn and Sn atomic concentrations alternated throughout the nanolaminated ZnO–SnO_2_ film having a ZnO surface layer. Under a sputter depth of 1600 s of etching, Zn rapidly faded and was substituted by Sn, indicating an interface between the layers. From the cyclic voltammetry measurement, the electrochemical reactions for intermixed and nanomixed ZnO–SnO_2_ and ZnO anodes can be described in the following equations:

Conversion: SnO_2_ + 4Li^+^ + 4e^−^ → Sn + 2Li_2_O

Alloying–dealloying: Sn + xLi^+^ + xe^−^ ↔ Li_x_Sn (0 ≤ x ≤ 4.4)

Conversion: ZnO + 2Li^+^ + 2e^−^ → Zn + Li_2_O

Alloying–dealloying: Zn + yLi^+^ + ye^−^ ↔ Li_y_Zn (0 ≤ y ≤ 1)

Although the CV curves of the intermixed and nanolaminated ZnO–SnO_2_ composites were similar, in the cathodic scan, the latter showed a lower reversibility than the former. Compare to single ZnO and SnO_2_ anodes, both intermixed and nanolaminated ZnO–SnO_2_ composites have a higher capacity and initial Coulombic efficiency (CE). The first discharge capacity and Coulombic efficiency (CE) of the intermixed ZnO–SnO_2_ were 2667 mAh g^−1^ and 80.2%, respectively, and for the nanolaminated ZnO–SnO_2_, these were 2471 mAh g^−1^ and 71.4%, respectively. The higher CE indicated that intermixed ZnO–SnO_2_ is more reversible. Both the intermixed and nanolaminated ZnO–SnO_2_ composites presented a better cycling stability than pure SnO_2_ and ZnO. For the nanolaminated composite, a capacity decrease occurred only after the 35th cycle. In comparison, the capacity of the intermixed composite was initially stable until the 10th cycle, subsequently increased to 1752 mAh g^−1^, and remained almost constant up to the 50th cycle. After the 50th cycle, particle expansion occurred in the intermixed ZnO–SnO_2_ electrode without disruption of their interconnections, resulting in a surface with fewer crevasses and more cycling stability than the nanolaminated composite surface. At the atomic scale, when mixing ZnO and SnO_2_, ZnO reduction suppresses the alloying of Sn, and the produced reduced Zn^0^ also assists SnO_2_, which improves the morphological stability of the anode during the cycling process and increases its cyclability. For the nanolaminated composite, the interface formation between ZnO and SnO_2_ leads to isolation intercalation and alloying reactions with the injected lithium, resulting in a severe volume change, specifically in the latter reaction. Moreover, the conductivity of the intermixed composite was improved. In view of these results, Zhao et al. (2019) explored the atomic ratio effect on the electrochemical properties of intermixed composites. For intermixed ZnO−2SnO_2_ composites containing ZnO and SnO_2_ in a 1:2 atomic ratio, the discharge capacities in the first, second, and thirtieth cycles were 2637, 2230, and 1771 mAh g^−1^, respectively, and the initial CE was 84.5%. The corresponding capacities for intermixed 2ZnO–SnO_2_ composites with ZnO and SnO_2_ in a 2:1 atomic ratio were 2495, 2018, and 1492 mAh g^−1^, and the first cycle CE was 80.9%. Both the ZnO–2SnO_2_ and 2ZnO–SnO_2_ composites had high cyclabilities of 1955 and 1794 mAh g^−1^ at 0.5 A g^−1^ after 50 cycles, respectively. It was concluded that the change in the atomic ratio did not significantly affect the electrochemical activity of these intermixed composites. Although the capacity could be tuned by changing the atomic ratio, the rate and cycling performance were minor. Interestingly, annealing the ZnO–SnO_2_ composite film in ambient helium at 1000 °C for 2 h led to the formation of a Zn_2_SnO_4_ film, making the surface of the annealed composite rough with some pinholes. The first and second discharge capacities of the Zn_2_SnO_4_ film were 2363 and 1915 mAh g^−1^, its CE was 81% after the first cycle, and its capacity remained ~1515 mAh g^−1^ at 0.5 A g^−1^ after 50 cycles. As the current density was increased in the order 0.5, 0.8, 1, 2, and 5 A g^−1^, the discharge capacity decreased in sequence as 1652, 1331, 1074, 818, and 514 mAh g^−1^. When the current density was reduced to 0.5 A g^−1^ again, the discharge capacity increased to reach 1448 mAh g^−1^, indicating the good rate capability of the Zn_2_SnO_4_ film. Thus, annealing did not have a significant influence on the electrochemical potential. Therefore, the most influencing factor of the electrochemical performance of ZnO–SnO_2_ composites is the degree of mixing, and not the crystallinity degree or the exact composition [6].

In addition to ZnO and SnO_2_, nickel oxide (NiO) is an interesting MO owing to its high theoretical specific capacity, low cost, eco-friendliness, and good electrochemical activities [38]. SnO_2_ undergoes reversible alloying–dealloying reactions, while NiO undergoes reversible conversion reactions. Transition MOs with unique structures such as multi-layer or yolk–shell structures present enhanced electrochemical performance [39,40]. Nanoscale materials provide enhanced surface areas compared to their bulk forms and thus contribute additional active sites for Li^+^ insertion/desertion, ensuring rapid transfer of charged particles [41,42]. Concurrently, a yolk–shell structure with a hollow feature provides a short pathway for Li^+^ and electron mobility, enhancing the kinetics of the electrochemical reaction [32,39]. Additionally, the presence of abundant channels on the porous shell allows important electrode/electrolyte interactions. Moreover, the hollow structure and the gap between the yolk and the shell can significantly prevent volume change and damage of the yolk–shell structure during the discharge/charge process [43,44,45]. Li et al. (2018) produced yolk–shell ZnO/NiO microspheres with a shell of nanorods and a microsphere yolk by a controlled thermal treatment of a bimetallic organic framework in air. Scanning electron microscopy (SEM) and high-resolution transmission electron microscopy (TEM) showed that the obtained microspheres had a diameter of 2 µm. There was a gap (~200 nm) between the yolk and the shell structure, which provided an additional barrier against the changes in the volume and structure during the cycling process [45]. The co-existence of Zn^2+^ and Ni^2+^ was critical for obtaining the yolk–shell structure. Without the introduction of the Ni^2+^ source (Ni(NO_3_)_2_●6H_2_O), only ultrathin products were produced after the solvothermal process, and ZnO nanoparticles (NPs) with diameters ≤ 100 nm were obtained after calcination. In contrast, without the injection of the Zn^2+^ source (Zn(NO_3_)_2_●6H_2_O), only layer stacking was achieved, and ultrathin NiO nanosheets were formed after calcination. The ZnO/NiO microspheres (0.276 g cm^−3^) had a higher packing density than commercial graphite (0.198 g cm^−3^), and a high-packing density electrode material is expected to result in high-energy density LIBs [46]. Thus, a large surface area along with mesopores can provide an additional barrier to penetration and prevent structural variation during the charge/discharge process. XPS analysis showed that the presence of amorphous carbon can increase the ZnO/NiO conductivity as well as offering a barrier to prevent structural change during the charge/discharge cycles [47]. The CV curves and X-ray powder diffraction (XRD) measurement indicated the reversible formation of ZnO and NiO after charging:

Conversion: ZnO + 2Li^+^ + 2e^−^ ↔ Zn + Li_2_O

Alloying–dealloying: Zn + xLi^+^ + xe^−^ ↔ Li_x_Zn

Conversion: NiO + 2Li^+^ ↔ Ni + Li_2_O

Due to the presence of NiO, the capacity of the composites is much higher than single ZnO and NiO. The first discharge/charge capacity of the ZnO/NiO microspheres was 1221.7/769.2 mAh g^−1^, and their initial CE was 62.9% at 0.1 A g^−1^. However, unlike the mentioned ZnO–SnO_2_ composite, the presence of NiO did not improve the initial CE. Therefore, different performances of the obtained composites can be found depending on the chosen MO partner. The electrolyte decomposition, SEI formation, occurrence of unclear irreversible reactions, and Li^+^ confinement in the electrode explain the low CE. The authors suggested that electrolyte optimization can enhance the initial CE of the MOs [48,49]. After 200 cycles, the specific capacity of the microspheres remained approximately 1008.6 mAh g^−1^. The ZnO/NiO microspheres demonstrated long-term cycling stability (592.4 mAh g^−1^ at 0.5 A g^−1^ after 1000 cycles) and a high rate capability (437.1 mAh g^−1^ at 2 A g^−1^). The high electrochemical activity of the ZnO/NiO microspheres can be explained based on their morphology and the ZnO and NiO synergetic effect. Additionally, the specific capacity of the ZnO/NiO microspheres increased during the charge/discharge cycles. For MO-based electrodes, the increase in the specific capacity can be attributed to the following: (1) generation of a polymeric layer on electrolyte degradation, which is called “pseudo-capacitance-type behavior” [50,51], (2) high accessibility of the host materials for Li^+^ insertion/desertion originating from the enhanced kinetics of Li diffusion by the activation process [52], (3) formation of defects in the charge/discharge process, which improves the reaction kinetics and contributes additional active sites for Li^+^ insertion/desertion [53], and (4) storage of interfacial lithium [54]. Electrochemical impedance spectroscopy (EIS) measurements showed that the charge transfer resistance (R_ct_) decreased on charge/discharge cycling, which can be ascribed to the following reasons: (1) improved charge particle mobilization owing to formation of the yolk–shell structure and amorphous-phase carbon composite [55,56], (2) increased electrolyte penetration in the electrode by the electrochemical reactions, which reduces the interfacial electrode/electrolyte impedance [38,51], and (3) enhanced Li^+^ diffusion kinetics owing to the repeated lithiation/delithiation on cycling. At 0.5 A g^−1^ and the 1000th cycle, the ZnO/NiO microsphere electrode still delivered a reversible capacity of 592.4 mAh g^−1^; however, some minor pulverization was observed. In addition, both capacitive and diffusion-controlled processes occurred in the ZnO/NiO electrode. The diffusion-controlled processes, i.e., intercalation, conversion, and alloying, resulted in the high capacity of the electrode, whereas the capacitive behavior ensured rapid charge movement [57,58]. The optimized temperature for the preparation of the ZnO/NiO microspheres was 450 °C (heating rate: 2 °C min^−1^) for 20 min [59]. 

Recently, Tu et al. (2021) introduced carbon cloth with a WO_3_/ZnO heterostructure film as an anode for LIBs. Tungsten trioxide (WO_3_) is another candidate to combine with ZnO to form WO_3_/ZnO composites. WO_3_ has a high theoretical capacity (~700 mAh g^−1^) [60,61] and similar limitations to other MOs such as quick capacity fading at a high current density, aggregation, and low conductivity. The carbon cloth (CC)-supported WO_3_/ZnO was prepared via the repeated immersion of CC-supported WO_3_ (previously prepared by hydrothermal and thermal treatment) into a ZnO QD solution, dried, and calcinated at 450 °C in air for 1 hour. Amorphous ZnO QDs (~2 nm) were found to be uniformly deposited on the surface of CC-supported WO_3_. According to the CV curve and XRD, this WO_3_/ZnO electrode undergoes both intercalation and conversion reactions during the discharge process. The obtained composite has a high discharge capacity (~1500 mAh g^−1^ at 0.28 *C*), rate performance (~500 mAh g^−1^ at 9.0 *C*), and cycling stability (1100 mAh g^−1^ at 1 *C* after 300 cycles). The high rate performance of the composites can be explained by the fast ion transfer due to the formation of the directional internal electric field at the interface of WO_3_ and ZnO in the WO_3_/ZnO heterostructures. In addition, the initial CE of the composites is 79.9%. However, the thick ZnO QD layer can lead to a decrease in the electrochemical performance due to the aggregation of ZnO into the gaps between WO_3_. The R_ct_ of WO_3_/ZnO is lower than that for WO_3_, thus confirming that the combination of WO_3_ and ZnO can increase the interfacial charge transfer kinetics. The increase in the reversible capacity of the WO_3_/ZnO electrode can be explained by both diffusion-controlled and surface-controlled processes, with a greater contribution belonging to the latter. The surface-controlled process resulted in the rapid lithiation/delithiation process at high rates, high stability, and high reversibility [62].

In another study, Karunakaran et al. (2018) synthesized a ZnO/Cu_2_MgO_3_ hollow porous nanocage as an anode for LIBs using a one-step cost-effective ultrasonic spray pyrolysis method. The nanocage had a diameter of ~400–700 nm, specific surface area of 46.373 m^2^g^−1^, pore size of 2–6 nm, and pore volume of 0.103 cm^3^ g^−1^. The porous structured shell resulted in easier electrolyte diffusion and more rapid Li^+^ transport and provided a buffer matrix to relieve the volume change during the cycling. The first discharge/charge capacity of the ZnO/Cu_2_MgO_3_ electrode at 0.3 A g^−1^ was 990.75 mAh g^−1^/663.45 mAh g^−1^, and the initial CE was 66.98%. The as-prepared composite anode had a high discharge capacity (528 and 441 mAh g^−1^ after 400 cycles at 0.3 and 0.5 A g^−1^, respectively), cycle stability, and rate capability. The charge transfer resistance of an as-assembled cell with ZnO/Cu_2_MgO_3_ as the anode was ~170 Ω, which decreased to approximately 146 Ω after four cycles, indicating a fading process after the cycling [63]. Field emission SEM images of the ZnO/Cu_2_MgO_3_ surface after 400 cycles at 0.5 A g^−1^ showed its robust and stable microstructure [64].

Similar to the yolk−shell structure composites, the composites of micrometer/sub-micrometer dimensions are promising as anodes for LIBs. Hou et al. (2015) synthesized hierarchical mesoporous ZnO/ZnFe_2_O_4_ (ZZFO) sub-microcubes (SMCs) as LIB anodes. The electrochemical reaction of ZnFe_3_O_4_ can be described by the following equation [19]:

Conversion: ZnFe_2_O_4_ + 9Li^+^ + 9e^−^ ↔ LiZn + 2Fe + 4Li_2_O

**Figure 3:**
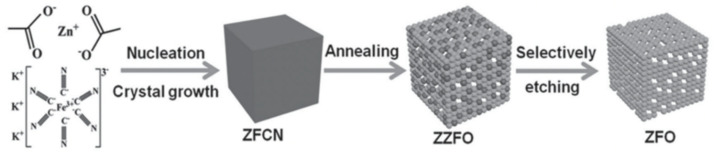
Scheme of ZZFO and ZFO synthesis process. Reprinted with permission from [71]. Copyright 2015, John Wiley and Sons.

ZZFO was prepared by post-calcination (500 °C for 2 h in air) of the Prussian blue analog (PBA) of Zn_3_[Fe(CN)_6_]_2_. Prussian blue and PBA can be employed as templates for preparing porous transition MOs [65,66,67,68,69]. X-ray fluorescence spectrometry confirmed that the ZnO/ZZFO molar ratio in the composite was 2:1. A simple chemical etching of ZZFO using 6 M NaOH for 5 h formed ZnFe_2_O_4_ (ZFO) (Figure 3). NaOH was used in the etching process because of ZnO dissolution in high-pH aqueous solutions [70]. The obtained products were cube-shaped, with dimensions in the range of ~500–700 nm. The ZZFO SMCs were composed of 10–15 nm NPs and contained many pores of ~3–7 nm between neighbor nanocrystallites. The results indicated that the ZZFO SMCs had a better initial discharge and reversible capacity, cycling performance, and rate capability than the single ZFO SMCs. The first discharge capacities of ZZFO and ZFO were ~1892 mAh g^−1^ and 854 mAh g^−1^, respectively, at 1 A g^−1^, and their initial CEs were ~70% and ~72%, respectively. In addition, at 1 A g^−1^, the discharge capacities of ZZFO and ZFO remained stable after 200 cycles. The good cycling properties of ZZFO and ZFO can be partially explained by the porous SMC structure, which provides additional free space against the volume change occurring during the repeated Li^+^ insertion/extraction process. Although ZFO had a higher specific surface area (SSA) than ZZFO, it had a lower specific capacity than ZZFO. Thus, it could be concluded that the Li storage performance of the ZZFO SMCs is dependent on the structure and the components, instead of the SSA. The electrochemical performance of the ZZFO SMCs can be attributed to the synergistic effect between ZnO and ZFO as well as the good dispersion of these nanophases. The homogeneous dispersion of ZnO prevents the self-aggregation of ZFO during the charge/discharge cycles and enhances the cycling performance of ZZFO. In addition, the ZnO and ZFO composite relieves the volume expansion during the charge/discharge process [71]. 

### 2.2. ZnO–Carbon-Based Composites

Forming MO composites, such as ZnO, with porous nanostructured carbon materials can enhance the specific capacity, mechanical stability/flexibility, and electronic conductivity of the MOs [7]. The carbon layer/matrix enhances the performance of MO LIB anodes by two mechanisms: (1) by serving as a buffer matrix to relieve the volume expansion, preventing its pulverization, and increasing the electrical conductivity [72,73,74,75], and (2) by offering additional beneficial effects such as increased Li storage sites, high electrical conductivity, and improved electrode/electrolyte wettability, leading to an enhanced specific capacity, cycling stability, and rate performance [76]. However, in many composites, because the porous carbon (PC) material can account for >50% of the total electrode weight, it only slightly improves the capacity. Consequently, the total energy density is significantly reduced, and PC materials are not highly suitable for actual LIB applications [77].

#### 2.2.1. ZnO-Coated Carbon-Based Material Composites

As mentioned above, porous nanostructured carbon-based materials can improve the specific capacity, cycling stability, and rate performance of MO anodes. They also offer a good conductive network and a buffer matrix for strain accommodation [5]. Hsieh et al. (2013) anchored ZnO nanocrystals (80–100 nm) with graphene nanosheets (GNs) using a method consisting of microwave heating, the modified Hummers method, and dispersion homogenization. A uniform dispersion of ZnO nanocrystals was found on both sides of the GNs and even in their interspacing layers. These ZnO nanocrystals not only acted as spacers to support the stereo GN framework but also as reduction–oxidation sites for improving the lithium storage capacity. In addition, the improvement in the rate capacity can be explained by the increase in the interlayer distance (i.e., *d_002_*) of the GNs due to the insertion of the ZnO nanocrystals. A large *d_002_* value yielded a high Li^+^ diffusion rate in the 3D GN framework. This can be explained by the migration of Li^+^ ions to bond to these sites as well as the active sites. When there were no ZnO spacers, the GNs tended to stack to form aggregates, decreased the accessible sites for the accommodation of Li^+^, and reduced the ionic diffusion rate performance [78]. The GNs served as a buffer against the volume change related to the strain during cycling [79], thereby increasing the cycling performance. Thus, the Zn@GN anode presented an improved performance compared to ZnO, with a Li storage capacity of 850 mAh g^−1^ (at 0.1 *C*), an 82% CE (at the first cycle), a good rate capacity (capacity retention ~60% at 5 *C*), and increased cycling stability (capacity decay ~8% at the 50th cycle at 1 *C*). In comparison, bare ZnO had a storage capacity of 606 mAh g^−1^ (at 0.1 *C*), a 48.4% CE (at first cycle), and a relatively poorer cyclic performance [20].

Owing to its nanoporous structure, good conductive network, and strain accommodation, PC has been utilized as a dispersion medium for many MOs, such as ZnO, SnO_2_, TiO_2_, and Fe_3_O_4_ [5,80,81]. It protects MOs from aggregation and pulverization [5]. Shen et al. (2013) prepared ZnO/PC composites using the solvothermal method. The first discharge/charge capacity of ZnO (54 wt%)/PC was 2017.4/1062.9 mAh g^−1^ (CE: 52.68%), and it had a high reversible capacity of 653.7 mAh g^−1^ at 0.1 A g^−1^ after 100 cycles. The high lithium storage potential of this ZnO/PC composite can be attributed to its nanoporous structure and interconnected network. Lowering the ZnO loading reduced the discharge capacity of the ZnO/PC composite, whereas increasing the ZnO loading blocked the PC pores. In addition, at a high ZnO loading, the increase in the ZnO particle size increased the mechanical stress in the composite. The morphology of pure ZnO severely deteriorated after the lithiation/delithiation cycling, which led to a low reversible capacity. In contrast, the ZnO/PC composite maintained its original morphology without significant pulverization or cracks after the charge/discharge cycles. It can be concluded that the PC host provided space for the volume variation in the ZnO particles during the intercalation/deintercalation process, thereby preventing electrode degradation [5].

Carbon fibers (CFs) can also be used as supporting and conducting materials [82]. Han et al. (2019) prepared CFs@pore–ZnO as anodes for LIBs. The shell thickness of ZnO was approximately 100 nm. After thermal treatment, the ZnO particles became porous and their surface was rougher, which can be attributed to the thermal decomposition of the carbon material in the metal–organic framework structure and the emission of CO_2_ and H_2_O. The rough surface of the CFs@pore–ZnO composite increased the electrode/electrolyte contact area, whereas the induced porosity was beneficial for Li^+^ mobility, electrolyte penetration, and addition of space against the volume expansion during the repeated Li^+^ insertion/desertion. Accordingly, the first discharge/charge capacities of the CFs@pore–ZnO composite and pure CFs were 955/533 mAh g^−1^ and 230/221 mAh g^−1^ at 0.1 A g^−1^, respectively. At the 300th cycle, the reversible capacity of the CFs@pore–ZnO composite was approximately 510 mAh g^−1^, whereas the discharge capacity of the pure CFs gradually reduced to 149 mAh g^−1^ after only 150 cycles. The discharge capacity of the composite was even higher than the estimated theoretical capacity, which can be explained by the synergistic effect between ZnO and the CFs as well as the Li^+^ storage in the voids between the ZnO polyhedra. The CFs@pore–ZnO composite exhibited good rates at 0.1, 0.2, 0.5, 1, and 2 A g^−1^, with discharge capacities of 462, 378, 313, 270, and 240 mAh g^−1^, respectively. In addition, when the current density was again decreased to 0.1 A g^−1^ after 60 cycles, the discharge capacity increased to 451 mAh g^−1^, which gradually increased to 510 mAh g^−1^ after 100 cycles. After 50 cycles, the pure CFs were pulverized, which did not occur in the CFs@pore–ZnO composite [83]. 

Three-dimensional graphene aerogels (GAs) have different applications such as catalysis, gas chromatography, gas storage and separation, and sensing, owing to their mechanical stability and high mass and electron transfer rates [84]. Fan et al. (2016) prepared GAs with anchored sub-micrometer mulberry-like ZnO (ZnO@GAs) as anodes for LIBs using the solvothermal method. In the XRD patterns, the diffraction peak at approximately 2θ = 25° disappeared, indicating the distribution of ZnO on both sides of the GA and prevention of stacking. The SEM images indicated that in the ZnO@GA composite, ZnO had a uniform diameter of ~500 nm and was composed of many small particles that aggregated into a mulberry-like morphology. Brunauer–Emmett–Teller (BET) analysis showed that the ZnO@GA composite had an SSA of 45.3 m^2^ g^−1^ and a well-defined 10.7 nm mesopore. These nanoporous voids are beneficial for the rapid diffusion of the electrolyte to the active sites. XPS analysis suggested the presence of C–O−Zn linkages (285.6 eV in C 1s and 532.1 in O 1s). Their formation can be attributed to the substitution of hydrogen in the hydroxyl groups or a possible ring-opening reaction of the epoxy groups by Zn^2+^ in ZnO [85,86,87]. The first discharge/charge specific capacity of the ZnO@GA composite was 1001/713 mAh g^−1^ at 1.6 A g^−1^ (initial CE: 71.2%). The reversible capacities of the as-prepared composite were 365, 320, and 230 mAh g^−1^ at 1, 2, and 10 A g^−1^, respectively. At 1.6 A g^−1^ and after 500 cycles, the reversible capacity remained at approximately 445 mAh g^−1^, with a CE of approximately 100%. The morphology of the nanoclusters was well preserved, and the spherical structure was clearly retained. The robustness of the spherical structure can be explained by the generation of an SEI layer around the polymer/gel-like coating, which maintains the structural integrity and thus enhances the electrochemical performance. EIS measurements confirmed that the ZnO@GA electrode had smaller charge transfer and interface layer resistances than the ZnO electrode. The high electrochemical performance is contributed by the strong oxygen bridges (C–O–Zn) resulting from the interaction of graphene and ZnO, providing a good pathway for electron transfer during the cycling process. In addition, the hierarchical structure of the ZnO microballs prevents the stacking of the graphene layers, allowing the GAs to facilitate Li^+^ transfer. The GA framework can also increase the electrical conductivity and relieve the electrode volume variation. The above synergistic effects improve the electrochemical performance [87].

The specific capacity of LIBs can be increased by the preparation of freestanding composites. In this type of composite, the use of binders and current collectors is rendered unnecessary [88]. It should be noted that a high mass loading is beneficial for a high energy density [77]. Nevertheless, ZnO-based composites frequently have low ZnO mass loadings (<1 mg cm^−2^) [89]. Concurrently, a thick electrode with a high mass density typically leads to a poor electrochemical performance owing to the high charge transport resistance and the electrode removal from the current collector [90,91,92]. To overcome these limitations, inactive materials, such as conductive agents, binders, and metallic current collectors, are required [77]. Therefore, composites with a high mass loading and that avoid inactive additives while maintaining their high electrochemical properties should be developed. In addition, the 3D structure of composites enhances the effective electrode surface area and promotes the diffusion of lithium ions [88]. Using this approach, Zhao et al. (2018) synthesized a composite of a 3D interconnected carbon foam anchored with a two-dimensional (2D) ZnO nanomembrane (C/ZnO NM foam) as an anode for LIBs. The advantage of a 2D NM is that its deformation to a wrinkled structure allows remarkable strain accommodation during lithiation without damage, such as cracking [12,93,94]. Concurrently, a 3D interconnected structure offers sufficient voids for high mass loading, prevents peeling off from thick active materials, and eliminates the utilization of organic binders [95,96]. In the above study, the carbon foam had a pore diameter of 200 µm and a surface area of ~50 m^2^ g^−1^, and it was freestanding and could be folded or compressed. Owing to the large contact area, ZnO NMs could be anchored on the carbon foam surface by physisorption. The composite presented a high surface area originating from the high ZnO loading as well as the high porosity of the carbon foam. The as-prepared anode maintained 92% capacity at 2 A g^−1^ and 5 A g^−1^ after 700 and 500 cycles, respectively, and achieved an areal capacity of 4.3 mAh cm^−2^ at 80 mA g^−1^, which is close to the acceptable capacity for practical applications (4 mAh cm^−2^) [77]. The C/ZnO NM foam realized discharge capacities of 450, 375, 288, 175, and 80 mAh g^−1^ at 0.25, 0.5, 1, 2, and 4 A g^−1^, respectively. When the current density was returned to 0.25 mA g^−1^, a capacity of ~450 mAh g^−1^ was recovered. After the first and second cycles, the CE was maintained at approximately 99.5%. This can be attributed to the role of the carbon foam structure of providing sufficient voids for the ZnO NM to release the strain by deformation without cracking. The carbon foam also prevents the generation of an SEI layer during the following cycles. The interconnected framework and the open pores allow carbon to enhance the electron and ionic transport over the electrode, assisting lithiation/delithiation reversibility. After 100 cycles at 0.64 A g^−1^, the starting structure of the carbon foam remained intact, and ZnO continued to be tightly anchored to the carbon foam framework. In contrast, the CE of the pure ZnO NMs was low because of the ZnO fracture and excessive formation of the SEI layer. To use C/ZnO NM foam anodes in full cells, cathode additives or extra cathodes are necessary to recover the capacity losses due to the SEI generation, which will sacrifice the energy density of the battery. In addition, specially designed electrolytes, such as gel-like electrolytes, and surface modification by thin oxides are effective approaches for improving the initial CE [77].

Li et al. (2017) formed another new freestanding composite, ZnO NM/expanded graphite (EG), using ALD. EG is a 3D material with an ultra-high SSA and large pores [97,98,99]. The large pores in EG provide sufficient voids for Li^+^ storage as well as volume expansion [100,101,102,103,104,105]. EG also provides mechanical support and conductive channels for active materials such as ZnO, thereby avoiding the electrode pulverization originating from the volume change during the cycling process. In the above study, the thickness of the ZnO layer was controlled by adjusting the ALD cycles (from 100 to 800 cycles). ALD prepares thin films by a surface chemical reaction. To prevent the occurrence of gas-phase reactions, precursors with surface-saturating concentrations are alternatively pumped into the reaction chamber. ALD achieves a conformal growth of NPs with precise thickness control and good adhesion. ZnO NM/EG could be compressed into a flexible and self-standing film and be embedded into a battery, without the requirement of a conductive agent, binder, and current collector. The resulting anode had a capacity of 438 mAh g^−1^ at 0.2 A g^−1^ after 500 cycles. The combination of the high capacity of ZnO and the support provided by EG explained the performance of the anode [106]. 

#### 2.2.2. Carbon-Based Material-Coated ZnO Composites

Coating carbon on ZnO leads to rapid electron mobility over its entire surface during cycling, thus enhancing the reversibility and kinetics of the Li^+^ insertion/extraction [107,108]. Carbon coating also protects MOs from dissolving in the electrolyte, protects composite deformation, and maintains high conductivity [7,109]. In 2016, Quartarone et al. synthesized graphite-coated ZnO nanosheets as binder-free anodes for LIBs. The ZnO nanosheets were prepared by the hydrothermal process and coated with graphite by thermal evaporation. The graphite-coated ZnO nanosheet composite having a graphite thickness of 350 Å presented the first discharge/charge capacity of 1470/968 mAh g^−1^ (CE: 65.85%) and a specific capacity of 600 mAh g^−1^ after 100 cycles at 1 A g^−1^. The specific capacity of the uncoated ZnO nanosheet was 400 mAh g^−1^ at 1 A g^−1^ after 100 cycles. The formation of micropores (pore diameter < 1 nm) within the composite and the enhancement of the exposed surface area were ascribed to the ZnO NPs’ nucleation. The ZnO NPs were 15 nm in size, and the nanosheet width and length were approximately 0.8–1.5 µm. The small sizes of the ZnO NPs and their nanostructure not only increased the electrode/electrolyte contact area and the electrical contact but also achieved high strain accommodation. Concurrently, the graphite coating acted as a buffer against the change in the composite morphology during the charge/discharge process [110].

**Figure 4:**
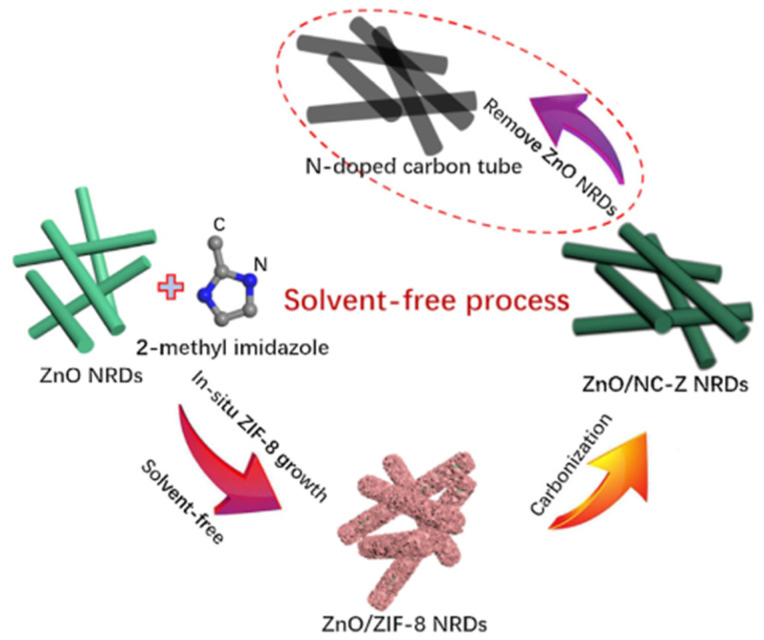
Scheme of synthesis of ZnO/NC-Z NRDs and N-doped carbon nanotubes. Reprinted with permission from [29]. Copyright 2017, Elsevier.

Gan et al. (2017) prepared N-doped carbon-coated ZnO nanorods (ZnO/NC-Z NRDs) using a solvent-free method as anodes for LIBs. N-doped carbon was synthesized by thermal treatment of zeolitic imidazolate framework-8 (ZIF-8), which was previously in situ grown on ZnO NRD surfaces (Figure 4). The study claimed that by using the in situ strategy, the adhesive force from the interaction between the carbon and ZnO NRDs was stronger than that with conventional ex situ methods, which resulted in a limited confinement effect in the latter [29]. Thus, compared to conventional ex situ methods, in situ methods realized a tighter coating of carbon on the ZnO NRD surfaces. TEM results showed that the carbon layer thickness was approximately 15–20 nm. Based on the XRD patterns, ZIF-8 was completely converted into amorphous carbon and ZnO after carbonization in Ar atmosphere. From the XPS analysis, the nitrogen concentration in the ZnO/NC-Z NRDs was approximately 2.3% and considered to enhance the composite electrical conductivity [29]. The carbon prepared from ZIF-8 exhibited specific properties such as a large surface area, tunable porosity, and structural stability and flexibility [29]. Owing to these characteristics, ZIF-8 can be employed in different energy storage systems, including supercapacitors, LIBs, and sodium-ion batteries [111,112,113,114]. Similar to the abovementioned carbon-based materials, N-doped carbon increased the NRD conductivity and, consequently, enhanced the Li^+^ diffusion rate. It also acted as a buffer layer, alleviating the volume variation during the cycling process. ZnO/NC-Z NRDs had a specific BET surface area of 135.9 m^2^ g^−1^ (higher than ZnO NRDs, 47.9 m^2^ g^−1^) and a mesoporous structure (pore size of 3.85–5.53 nm). The presence of N-doped carbon also increased the ZnO/NC-Z NRD pore volume. The enhancements in the SSA and pore volume can be attributed to the decomposition of the ZIF-8 framework during the sintering process. The as-prepared composite had a capacity of 1439 mAh g^−1^ at 0.2 A g^−1^ and an initial CE of 76%; the latter was higher than that of the ZnO/C-P NRDs (71%) (sample prepared by ex situ method) and ZnO NRDs (67%). The higher CE of the ZnO/NC-Z NRD composite can be explained by the coated carbon layer preventing the detrimental reactions between the electrolyte and the ZnO NRDs. After 200 and 850 cycles, the capacity of the composite was reduced to 1011 mAh g^−1^ at 0.2 A g^−1^ and to 544 mAh g^−1^ at 1 A g^−1^ (capacity retention: 87.7%). The CE of the ZnO/NC-Z NRDs was found to be approximately 100% after the first cycle, indicating the ease of Li^+^ insertion/extraction and efficient electron and ion transfer. Using the four-point probe method showed that the electrical conductivity of the ZnO/NC-Z NRDs was higher than that of the ZnO/C-P NRDs and ZnO NRDs. After 150 cycles, the NRD morphology of the ZnO/NC-Z NRDs was preserved without any significant structural damage. The charge transfer resistance (R_(sf+ct)_) of the ZnO/NC-Z NRDs (61 Ω) was lower than that of the ZnO/C-P NRDs (102 Ω) and ZnO NRDs (175 Ω). Moreover, the ZnO/NC-Z NRDs could be additionally used as precursors to synthesize N-doped carbon nanotubes (CNTs) (Figure 4), which had a capacity of 1001.1 mAh g^−1^ after 100 cycles at 0.2 A g^−1^ and a capacity retention of 99.1% [29].

Recently, Thauer et al. (2021) introduced ZnO/C composites prepared via thermal treatment. As anodes for LIBs, ZnO/C composites calcinated at 700 °C showed the first discharge/charge capacities of 1061/671 mAh g^−1^ at 0.1 A g^−1^ (initial CE: 63.24%). The carbon content in the optimized composite was found to be around 5.7 wt.%. XRD measurement confirmed the two-step reaction mechanism, including the conversion and alloying processes (Equations (1) and (2)). After the 100th cycle at 0.1 A g^−1^, the discharge capacity of the ZnO/C composite was reduced to 212 mAh g^−1^. The irreversible conversion reaction can explain the rapid capacity. The presence of carbon is believed to improve the electronic conductivity and Li^+^ diffusion [115]. Eisenmann et al. (2021) indicated that the carbon coating on Mn-doped ZnO could partially reduce manganese, reallocate the crystal structure, and increase the specific capacity of Mn-doped ZnO [116]. The particle size of carbon-coated Mn-doped ZnO and Mn-doped ZnO was similar (~ 20 nm), indicating that the carbon layer did not affect the size of particles. Through measurement with thermal gravimetric analyzers, the carbon content was ~20 wt.%. The lithium reaction mechanism was nearly the same between carbon-coated Mn-doped ZnO and Mn-doped ZnO. However, more reversible alloying–dealloying and much more reversible conversion were found in the case of carbon-coated Mn-doped ZnO. At 0.1 *C*, carbon-coated Mn-doped ZnO had higher specific capacities (~200 mAh g^−1^). The authors found that a carbon-coated layer is most likely to improve the capacity retention and rate capability rather than result in a capacity improvement. When the current density was increased from 0.1 to 1 *C*, the specific capacity decreased by only 138 mAh g^−1^ from 740 to 602 mAh g^−1^. Overall, the presence of a coated carbon layer can increase the electrochemical performance of Mn-doped ZnO via the decreased volume change upon cycling and the improvement in the reversible conversion reaction [116].

## 3. ZnO Ternary Composites

Besides binary composites, ternary composites have been investigated in different studies. Most of these ternary composites comprised ZnO, another MO, and PC-based materials. The synergistic effects between these materials can explain the improved electrochemical performance of the corresponding composites. For example, the PC materials serve as a buffer matrix to alleviate the volume expansion and capacity fading problems, whereas the ZnO and MO composite can protect the active materials from aggregation and enhance the electrode conductivity [117]. However, the electrochemical characteristics of ternary composites are less remarkable than those of the abovementioned binary composites.

Kose et al. (2016) prepared a freestanding ZnO/SnO_2_/multi-walled CNT (ZnO/SnO_2_/MWCNT) buckypaper composite using the sol–gel coating method. The MWCNT structure provided the mechanical stability offered by active materials. At the first cycle and 0.2 *C*, the specific capacities of ZnO/SnO_2_/MWCNT, ZnO/MWCNT, and SnO_2_/MWCNT were 1584, 1152, and 1491 mAh g^−1^, respectively, which, after 100 cycles, were reduced to 487, 460, and 441 mAh g^−1^, respectively. The MWCNTs act as a buffer matrix by interacting with the MO cluster and preventing the volume variation and capacity fading [118]. The higher discharge capacity of ZnO/SnO_2_/MWCNT than that of the binary composites originates from the compositing of ZnO and SnO_2_ as well as the high conductivity provided by the MWCNTs. In the binary composites, aggregation of the MOs is one of the significant problems [118,119]. In ZnO/SnO_2_/MWCNT ternary composites, the uniform dispersion of the MOs as well as the diffusion barriers between ZnO and SnO_2_ prevents the aggregation of Zn and Sn atoms [117]. Recently, Zhang et al. (2021) prepared SnO_2_/ZnO@Polypyrrole (PPy) via an electrospinning technology. SnO_2_ SnO_2_/ZnO was prepared with polyvinylpyrrolidone (PVP) and then coated with PPy via the electrospinning process. PVP with a high molecular weight (Mw = 1,300,000 g mol^−1^) resulted in a smaller diameter, dense structure, and higher electrochemical performance than PVP with a lower molecular weight (Mw = 58,000 g mol^−1^). The obtained composites had an initial discharge/charge capacity of 1861.8/1138.1 mAh g^−1^ at 0.2 *C* (initial CE: 61.12%). After 100 cycles at 0.2 *C*, the discharge capacity of the SnO_2_/ZnO@PPy composite was reduced to 626.1 mAh g^−1^. Ppy can increase the capacity of composites by enhancing the conductivity of the composites and alleviating the change in the electrode during electrochemical cycling [120,121,122,123]. Similar to porous carbon materials, the porosity of PPy also improved the contact area with the electrolyte, accelerated the ion/electron diffusion rate, and increased the Li^+^ reversibility during the cycling process. EIS measurement additionally indicated that SnO_2_/ZnO@PPy has a higher charge transfer rate than SnO_2_/ZnO due to the presence of PPy [124]. 

In addition to SnO_2_, SnO, which is formed by the reduction of SnO_2_, has a high theoretical capacity (approximately 880 mAh g^−1^) [125]. Joshi et al. (2016) examined binder-free SnO_x_–ZnO/carbon nanofiber (CNF) composites as LIB anodes. The optimal Sn/Zn ratio for the SnO_x_–ZnO CNF composites was found to be 75:25 (wt.%). Increasing the ZnO content decreased the electrochemical performance of the composite owing to its low electrochemical activity [126]. At the optimal condition, the first discharge/charge capacity of the SnO_x_–ZnO CNF composite was 1910/1400 mAh g^−1^ (CE: 73.3%) at 0.1 A g^−1^. After 55 cycles, its reversible capacity was 963 mAh g^−1^ at 0.1 A g^−1^. The CNFs enhanced the Li_2_O decomposition and thus increased the reversible capacity. In addition, ZnO protected Sn from aggregation, which resulted in a cell with a high discharge capacity and increased stability. Amorphous SnO_x_ and ZnO were embedded in the CNFs, and the morphology of the resultant SnO_x_–ZnO CNFs was uniform, smooth, long, and free of agglomerated particles. Moreover, there was no significant deterioration of the SnO_x_–ZnO CNF composite morphology after 55 cycles [127]. 

Another ternary composite can be formed with ZnO and NiO. Similar to binary ZnO/MO and SnO_2_/MO composites, the limitations of NiO/MO binary composites originate from their poor electronic conductivity and structural change during the repeated cycling, which result in a poor rate capability and rapid capacity fading. In 2018, Ma et al. prepared a NiO–ZnO/reduced graphene oxide (NiO–ZnO/RGO) composite by a process consisting of ultrasonic, freeze drying, and thermal treatments. The SEM results showed that the synthesized NiO–ZnO nanoflakes were uniformly distributed on the RGO sheet. The as-prepared electrodes had a first discharge capacity of 1393 mAh g^−1^ (CE: 66.3%) and high reversible capacities of 1017 mAh g^−1^ at 0.1 A g^−1^ after 200 cycles and 458 mAh g^−1^ at 0.5 A g^−1^ after 400 cycles. The reversible capacity of the ternary composite (1017 mAh g^−1^) was higher than the theoretical capacity (833 mAh g^−1^). After 15 cycles, the NiO–ZnO/RGO composite electrode had a higher CE (98%) than the NiO–ZnO hybrid anode. The reversible capacities of NiO and NiO–ZnO were reduced to 212 mAh g^−1^ at 180 cycles and 247 mAh g^−1^ at 150 cycles, respectively. The enhancement in the capacity of the NiO–ZnO/RGO composite was contributed by the formation of a reversible polymeric gel-like film with a high material viscosity provided by ZnO, which enhances the adhesion between the active material layer and the current collector [128]. NiO–ZnO/RGO had a smaller charge transfer resistance than the NiO–ZnO binary composite. Similar to MWCNTs and other carbon-based materials, the RGO prevents NiO–ZnO agglomeration and the volume variation during the charge/discharge cycles. NiO–ZnO nanoflakes were considered to provide abundant electrochemical reaction sites and decrease the Li^+^ diffusion length, whereas the role of RGO was to enhance the Li^+^ and electron transfer rates during the cycling process [129]. In the above study, the synergistic effect between NiO–ZnO and RGO was similar to that in the abovementioned binary composites. However, the combination effects of NiO and ZnO were not clearly explained.

**Figure 5:**
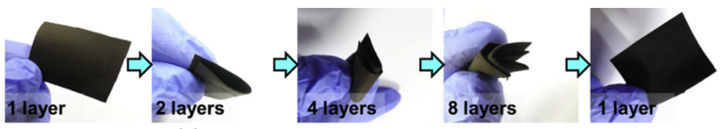
Folding process of FGCZ composite. Reprinted with permission from [133]. Copyright 2019, Elsevier.

Germanium oxide (GeO_2_) is a promising anode material because of its high theoretical reversible capacity (1125 mAh g^−1^), low operating voltage, and good thermal stability [130,131,132]. He et al. (2019) reported freestanding mesoporous foldable GeO_x_/ZnO/C (FGCZ) composite nanofibers with uniform distributions of GeO_x_ and ZnO. A solution of polyacrylonitrile (PAN), zinc acetate (Zn(Ac)_2_), CNTs, and GeO_2_ NPs was used to fabricate nanofibers by electrospinning. The precursor nanofibers were stabilized by 2 h of annealing in air at 250 °C and were subsequently carbonized by 6 h of annealing in air at 700 °C to yield the FGCZ nanofibers. The resulting nanofibers possessed uniform diameters of approximately 300 nm, longer than those of the GeO_x_ sample. The above can be explained based on the presence of Zn(Ac)_2_ causing plasticization of PAN via the formation of N–Zn coordinative bonds. XPS analysis confirmed the existence of Ge_2_O_3_, formed by the reduction of GeO_2_ by carbon at 700 °C, as well as ZnO in the composite fibers. Raman spectra, via I_D_/I_G_, indicated FGCZ was more disordered than GC (the sample prepared in the absence of Zn(Ac)_2_) owing to the presence of Zn(Ac)_2_. FGCZ also had a larger surface area (532.56 m^−2^ g^−1^) than GC (236.33 m^−2^ g^−1^). Mesopores with widths of 4–7 nm were also found in FGCZ, whose highly porous structure enhanced the electron transmission, provided more Li storage sites, and increased the rate of Li ion transport, thus improving the electrochemical performance. In their study, besides serving as the ZnO precursor and promoting the formation of mesopores, the added Zn(Ac)_2_ in the electrospun solution achieved the following: (i) enhancement in the mechanical properties and flexibility of the composites (Figure 5), and (ii) assisting in the dispersion of GeO_2_ NPs. Thus, the as-prepared FGCZ composite presented good electrochemical characteristics with a first discharge/charge capacity of 1000/890 mAh g^−1^ at 0.2 A g^−1^ (CE: 66.9%). After the 200th cycle at 0.2 A g^−1^ and 500th cycle at 1 A g^−1^, the FGCZ composite achieved discharge capacities of 617 and 464 mAh g^−1^, respectively. Owing to the presence of amorphous active materials, their uniform dispersion, and the good conductivity of CNTs, the FGCZ composite showed rapid Li ion diffusion and therefore exhibited higher reversible capacities than the GC sample at the same high current density. When the FGCZ composite was assembled into full cells using a commercial flexible LiCoO_2_/CNT as the cathode, it displayed a discharge capacity of 417 mAh g^−1^ at 0.1 A g^−1^. A ten-fold bent full battery had a discharge capacity of 391 mAh g^−1^, which confirmed the good mechanical stability of the FGCZ composite. He et al. (2019) found that their full battery with FGCZ powered a light-emitting diode under different bending conditions. Specifically, under different bending angles, there was no significant difference in the EIS plots of the FGCZ composite [133].

**Figure 6:**
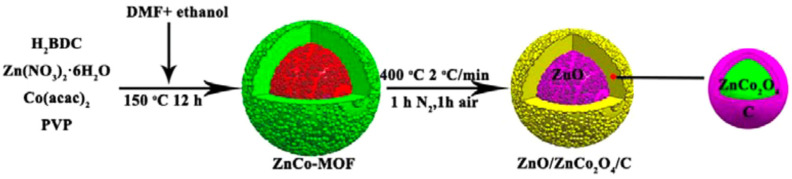
Scheme of preparation of ZnO/ZnCo_2_O_4_/C composite. Reprinted with permission from [28]. Copyright 2015, American Chemical Society.

Co_3_O_4_ is also considered as an anode candidate for LIBs owing to its high theoretical capacity [134]. However, it is limited by its high cost and toxicity [28]. To reduce its cost and improve its eco-friendliness, Co can be replaced by different metals, such as Fe, Mn, Ni, and Zn, to form a ternary structure [134]. Among the various combinations, ZnCo_2_O_4_ is a promising candidate because of its high specific capacity (975 mAh g^−1^), inexpensive cost, low operating voltage, and environmental friendliness [135,136,137,138]. Ge et al. (2015) synthesized a ZnO/ZnCo_2_O_4_/C PC/shell composite comprising ZnCo_2_O_4_ as the shell, ZnO as the core, and a homogeneously carbon-coated ZnCo_2_O_4_ shell surface (Figure 6). The ZnO/ZnCo_2_O_4_/C composite had a diameter of 800 nm and a mesoporous structure with an SSA of 27.9 m^2^ g^−1^, an average pore size of 14 nm, and a pore volume of 0.146 cm^3^ g^−1^. The initial discharge/charge capacity of the ZnO/ZnCo_2_O_4_/C composite was 1278.8/974 mAh g^−1^ at 0.5 A g^−1^, and the initial CE was 76.2%. The as-prepared ternary composite maintained a reversible capacity of 669 mAh g^−1^ after 250 cycles at 0.5 A g^−1^ and 715 mAh g^−1^ after 50 cycles at a high rate of 1.6 A g^−1^. In contrast, at 0.5 A g^−1^, the reversible capacity of the ZnO/ZnCo_2_O_4_ composite rapidly reduced to 524.4 mAh g^−1^ only after 110 cycles. The structure of the ZnO/ZnCo_2_O_4_/C composite was maintained well after 250 cycles, without any collapse and shedding. The EIS of ZnO/ZnCo_2_O_4_/C had a smaller diameter than that of ZnO/ZnCo_2_O_4_, indicating that the former had a smaller charge transfer resistance and more rapid reaction throughout the charging process owing to the existence of the carbon layer. Overall, hierarchically porous core/shell structures provide abundant active sites, improve the electrode/electrolyte contact area, and offer abundant channels for the penetration of the electrolyte. Moreover, they relieve the structure pulverization caused by the Li^+^ insertion/desertion. Concurrently, the carbon layer effectively enhances the composite conductivity, therefore improving the electron transfer rate, efficiently protects ZnCo_2_O_4_ from agglomeration and pulverization, and partially alleviates the strain resulting from the volume variation during the cycling process [28]. 

**Figure 7:**
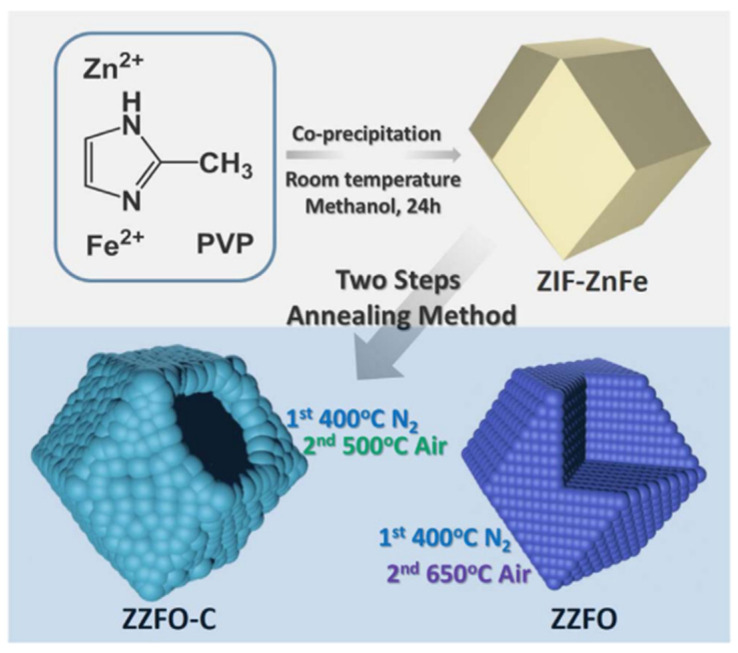
Scheme of synthesis process of ZZFO-C and ZZFO. Reprinted with permission from [142]. Copyright 2017, Elsevier.

Similar to ZnCo_2_O_4_, Ma et al. (2017) introduced ZnO/ZnFe_2_O_4_/N-doped C-micro-polyhedra (ZZFO-C) as anodes for LIBs. The composites were prepared by thermal treatment of ZIF–ZnFe (molar ratio of 3:1) for 2 h at 500 °C (Figure 7). XRD results confirmed the co-existence of ZnFe_2_O_4_ and ZnO. XPS analysis identified various nitrogen-doped carbon species and oxygen functional groups. The ZZFO-C composites had an average size of ~420 nm, slightly smaller than that of the ZIF–ZnFe precursor, owing to the partial framework decomposition and contraction throughout the calcination. The ZZFO-C composites possessed rougher surfaces than their precursors and comprised clustered MOs/carbon NPs (size of ~20 nm) and many small holes. The formation of the hollow structure is a consequence of the decomposition of the inner ZIF–ZnFe to form gaseous products, such as CO_2_, H_2_O, and NO_2_ [66,75,139,140,141]. The ZnO/ZnFe_2_O_4_ NPs and the carbon layers overlapped with each other in the composite. Ma et al. (2017) found that when the annealing temperature was increased to 650 °C, the carbon in the composite was removed (noted as ZZFO) (Figure 7). The surface area and pore size of ZZFO-C were 84.3 m^2^ g^−1^ and 13 nm, respectively, and for ZZFO, they were 31.2 m^2^ g^−1^ and 39 nm, respectively. The mesoporous structures of ZZFO-C and ZZFO were beneficial for the Li^+^ transport and could alleviate the volume change during insertion/desertion cycles. In terms of the electrochemical performance, ZZFO-C had a first discharge capacity of 1751 mAh g^−1^ (CE: 67.4%). The higher CE of ZZFO-C than that of ZZFO (60.8%) can be explained by the presence of the N-doped carbon matrix, which improves the reversibility of the electrode. After 100 cycles at 0.2 A g^−1^ and 2.0 A g^−1^, the obtained ZZFO-C composite presented reversible capacities of 1000 mAh g^−1^ and 620 mAh g^−1^, respectively. It also displayed a good rate capability with specific capacities of 1075, 1052, 1024, 928, 842, and 787 mAh g^−1^ at 0.05, 0.1, 0.2, 0.5, 1, and 2 A g^−1^, respectively. After returning to 0.1 A g^−1^ at the 75th cycle, the ZZFO-C composite recovered its initial specific capacity (1190 mAh g^−1^), which continuously increased to reach 1328 mAh g^−1^ at the 90th cycle. Compared to ZZFO-C, the ZZFO sample showed poorer electrochemical activity. The good electrochemical performance of the ZZFO-C composite can be attributed to the synergistic effect between the N-doped carbon matrix and the two active components as well as its distinct hierarchical hollow structure [142].

Zhang et al. (2016) prepared a freestanding 3D ZnO/graphene/CNT ternary composite as an anode for LIBs by the sol–gel technique followed by vacuum-assisted filtration. Based on thermogravimetric analysis, the ZnO NPs (average size of 8 nm) accounted for approximately 84 wt% and were uniformly distributed on the composite. The ZnO/graphene/CNT composite had a first discharge capacity of 1503 mAh g^−1^ at 0.1 A g^−1^ (CE: 60%) and high cyclability and rate capability, with a reversible discharge capacity of 620 mAh g^−1^ after 100 cycles at 0.1 A g^−1^. The CE was approximately 100% from the seventh cycle. Similar to the previously mentioned ZnO NPs and PC binary composites, the large surface area and high conductive network of the graphene/CNT structure maintain good electronic contact between the particles and suppress the aggregation and volume variation in the ZnO NPs during the cycling process. In addition, the CNTs not only adopt the role of a graphene modifier, change the surface characteristics, and prevent the agglomeration of active materials but also serve as a link between the graphene layers [88]. These effects contribute to the high first discharge capacity and good cycle/rate performance of the ternary composite [88].

## 4. Conclusions and Perspectives

ZnO is a promising anode candidate for LIBs owing to its high theoretical capacity (978 mAh g^−1^) [5]. However, because of its limitations, such as its slow chemical reaction kinetics, rapid capacity fading, and poor rate capability [20], composites of ZnO must be formed with other materials. The highlighted studies on ZnO-based binary and ternary composites with different synthesis methods are summarized in Table 2, Table 3. Most ZnO-based composites had higher first discharge capacities than only ZnO. Moreover, most of the composites discussed in this review showed a good cycling stability and rate performance. The large electrode/electrolyte contact area, abundant charge storage reaction sites, short Li^+^ diffusion path, improved conductivity, stability structure, and potential to relieve the volume expansion could explain the good electrical performance of these anode composites. To synthesize high-performance anodes, different factors such as size, morphology, crystallinity, phase composition, and porosity should be considered [9]. Some limitations of ZnO-based composites have also been found and should be overcome rapidly. The synergistic effects between the components of the composites as well as the correlation between the composite structure and the electrical performance should be investigated in more detail. In general, the initial CEs of ZnO-based composites were low and should be increased, for which electrolyte optimization, surface modification, and coating of ZnO are possible solutions [9,48,49]. In the case of ZnO–MO binary composites, the choice of MO partner to prepare ZnO–MOs may increase the initial CE. Both conversion and alloying MOs can increase the capacity of composites. However, between reversible conversion and alloying–dealloying reactions, the reversible alloying–dealloying reaction may have more benefits in the increase in the initial CE. For example, the presence of alloying materials (SnO_2_) in the composite can increase the initial CE, while the presence of conversion materials (NiO) has less impact on the improvement in the initial CE [6,59]. Moreover, the relationships between active materials, binders, and electrolyte additives should be further investigated in order to improve the SEI layer and thus result in a better CE and cycle performance [9]. Strategies to develop ZnO-based composites that have high energy densities should be identified. For example, in ZnO–carbon-based composites, the carbon component contributes slightly to the capacity but accounts for more than 50% of the total electrode weight. Therefore, the total energy density of these composites is significantly lowered, which reduces the potential of PC materials in real LIB applications. In addition, there are considerable efforts to synthesize ZnO ternary composites with different structures. However, their electrochemical performance is not significantly higher when compared to similar binary composite anodes for LIBs. For example, the ZnO/SnO_2_/MWCNT and NiO–ZnO/RGO ternary composites mentioned in this paper did not have a better initial discharge capacity and cycling performance when compared to ZnO–SnO_2_ and ZnO–NiO. In addition, most of the studies mentioned in this review focused on developing composites with a high specific capacity instead of producing composites with a high packing density and a high energy density. Thus, we recommend aiming at generating novel composites or optimizing ZnO-based composites that not only have a high specific capacity, cycling stability, and rate performance but also a high initial CE and energy density. In this review, some studies such as Li et al. (ZnO–NiO microspheres, 2018) and Zhao et al. (C/ZnO NMs, 2018) focused on improvements not only in the electrochemical performance of their studied composites but also in their energy density [59,77]. Moreover, a more synthetic process than the current ones should be developed to reduce the production cost to meet industrial requirements.

## Figures and Tables

**Table 1:** Common MOs utilized as LIB anodes and their theoretical capacities.

Metal Oxide	Lithium Intercalation Method	Theoretical Capacity (mAh g^−1^)	References
Co_3_O_4_	Conversion	890	[9,11]
CoO	Conversion	716	[11]
CuO	Conversion	674	[11]
Fe_2_O_3_	Conversion	1006	[12]
NiO	Conversion	718	[9,13]
RuO_2_	Conversion	806	[11,14]
SnO_2_	Alloying	740	[15]
TiO_2_	Intercalation	335	[9,16]
ZnCo_2_O_4_	Conversion	903	[9,17]
ZnFe_2_O_4_	Conversion	1000	[18,19]
ZnO	Alloying	978	[5,7,10]

**Table 2:** Summary of highlighted ZnO binary composite studies.

Anode	Synthesis Method	Electrochemical Performance				Reference
		Initial Discharge Capacity (mAh g^−1^)	Initial CE (%)	Cycling Performance (mAh g^−1^/Cycles)	Current Density	
Intermixed ZnO–SnO_2_	ALD	2667	80.2	1752/50	0.5 A g^−1^	[6]
Nanolaminated ZnO–SnO_2_	ALD	2471	71.4	~1250/50	0.5 A g^−1^	[6]
ZnO–NiO microspheres	Controlled calcination treatment	1221.7	62.9	1008.6/200	0.1 A g^−1^	[59]
WO_3_/ZnO film	Hydrothermal and thermal treatment	~1500	79.9	~1100/300 at 1 *C*	0.28 *C*	[62]
ZnO/Cu_2_MgO_3_	One-step cost-effective ultrasonic spray pyrolysis	990.75	66.98	528/400	0.3 A g^−1^	[64]
ZnO/ZnFe_2_O_4_ SMCs	Thermal treatment	1892	~70	837/200 cycles	1 A g^−1^	[71]
ZnO@GNs	High-performance homogenizing	850	82	Capacity decay of ~8% after 50 cycles at 1 *C*	0.1 *C*	[20]
ZnO/PC	Solvothermal	2017.4	52.68	653.7/100	0.1 A g^−1^	[5]
CF@pore-ZnO	Thermal treatment	955	55.81	510/300	0.1 A g^−1^	[83]
C/ZnO NMs	Pyrolysis and immersion coating	~1100	59	260/700 at 2 A g^−1^	0.08 A g^−1^	[77]
ZnO/EG	ALD	~1000	~60	438/500	0.2 A g^−1^	[106]
ZnO@GAs	Solvothermal	1001	71.2	445/500	1.6 A g^−1^	[87]
Graphene-coated ZnO nanosheet	Hydrothermal and thermal evaporation process	1470	65.8	600/100	1 A g^−1^	[110]
ZnO/NC-Z NRDs	Thermal treatment	1439	76	1011/200	0.2 A g^−1^	[29]
ZnO/C	Thermal treatment	1061	63.24	212/100	0.1 A g^−1^	[115]
Carbon-coated Mn-doped ZnO	Thermal treatment	~1200	~61.6%	~740/80	0.1 *C*	[116]

**Table 3:** Summary of highlighted ZnO ternary composite studies.

Anode	Synthesis Method	Electrochemical Performance				Reference
		Initial Discharge Capacity (mAh g^−1^)	Initial CE (%)	Cycling Performance (mAh g^−1^/Cycles)	Current Density	
ZnO/SnO_2_/MWCNT	Sol–gel coating	1584	~57	487/100	0.2 *C*	[117]
SnO_2_/ZnO@PPy	Electrospinning and thermal treatment	1861	61.12	626.1/100	0.2 *C*	[124]
SnO_x_–ZnO/CNF fiber	Electrospinning and thermal treatment	1910	73.3	963/55	0.1 A g^−1^	[127]
NiO–ZnO/RGO	Ultrasonic, freeze drying, and thermal treatment	1393	66.3	1017/200	0.1 A g^−1^	[129]
GeO_x_/ZnO/C	Electrospinning and thermal treatment	1000	66.9	617/200	0.2 A g^−1^	[133]
ZnO/ZnCo_2_O_4_/C	Thermal treatment	1278	76.2	669/250	0.5 A g^−1^	[28]
3D ZnO/graphene/CNTs	Sol–gel technique following by vacuum-assisted filtration	1503	60	620/100 cycles	0.1 A g^−1^	[88]

## Data Availability

Statistics about published articles/patents related to MOs and their composites for LIB applications are archived from Google Scholar (https://scholar.google.com) (accessed on 15 June 2021) database with keyword: “Metal oxide lithium-ion batteries”.

## References

[1] Kang S., Li Y., Wu M., Cai M., Shen P.K. (2014). Synthesis of hierarchically flower-like FeWO_4_ as high performance anode materials for Li-ion batteries by a simple hydrothermal process. Int. J. Hydrog. Energy.

[2] Nguyen Q.H., Phung V.D., Kidanu W.G., Ahn Y.N., Nguyen T.L., Kim I.T. (2021). Carbon-free Cu/Sb_x_O_y_/Sb nanocomposites with yolk-shell and hollow structures as high-performance anodes for lithium-ion storage. J. Alloys Compd..

[3] Zhu J., Zhang G., Gu S., Lu B. (2014). SnO_2_ nanorods on ZnO nanofibers: A new class of hierarchical nanostructures enabled by electrospinning as anode material for high-performance lithium-ion batteries. Electrochim. Acta.

[4] Li H., Wei Y., Zhang Y., Yin F., Zhang C., Wang G., Bakenov Z. (2016). Synthesis and electrochemical investigation of highly dispersed ZnO nanoparticles as anode material for lithium-ion batteries. Ionics.

[5] Shen X., Mu D., Chen S., Wu B., Wu F. (2013). Enhanced electrochemical performance of ZnO-loaded/porous carbon composite as anode materials for lithium ion batteries. ACS Appl. Mater. Interfaces.

[6] Zhao B., Mattelaer F., Kint J., Werbrouck A., Henderick L., Minjauw M., Dendooven J., Detavernier C. (2019). Atomic layer deposition of ZnO–SnO_2_ composite thin film: The influence of structure, composition and crystallinity on lithium-ion battery performance. Electrochim. Acta.

[7] Zhang J., Gu P., Xu J., Xue H., Pang H. (2016). High performance of electrochemical lithium storage batteries: ZnO-based nanomaterials for lithium-ion and lithium-sulfur batteries. Nanoscale.

[8] Aravindan V., Jinesh K.B., Prabhakar R.R., Kale V.S., Madhavi S. (2013). Atomic layer deposited (ALD) SnO_2_ anodes with exceptional cycleability for Li-ion batteries. Nano Energy.

[9] Chen Y., Chen X., Zhang Y. (2021). A Comprehensive Review on Metal-Oxide Nanocomposites for High-Performance Lithium-Ion Battery Anodes. Energy Fuels.

[10] Yuan G., Wang G., Wang H., Bai J. (2015). Synthesis and electrochemical investigation of radial ZnO microparticles as anode materials for lithium-ion batteries. Ionics.

[11] Fang S., Bresser D., Passerini S. (2020). Transition Metal Oxide Anodes for Electrochemical Energy Storage in Lithium- and Sodium-Ion Batteries. Adv. Energy Mater..

[12] Liu X., Si W., Zhang J., Sun X., Deng J., Baunack S., Oswald S., Liu L., Yan C., Schmidt O.G. (2014). Free-standing Fe_2_O_3_ nanomembranes enabling ultra-long cycling life and high rate capability for Li-ion batteries. Sci. Rep..

[13] Song Y., Hwang J., Lee S., Thirumalraj B., Kim J.H., Jenei P., Gubicza J., Choe H. (2020). Synthesis of a High-Capacity NiO/Ni Foam Anode for Advanced Lithium-Ion Batteries. Adv. Energy Mater..

[14] Hassan A.S., Moyer K., Ramachandran B.R., Wick C.D. (2016). Comparison of Storage Mechanisms in RuO_2_, SnO_2_, and SnS_2_ for Lithium-Ion Battery Anode Materials. J. Phys. Chem. C.

[15] Nguyen T.P., Kim I.T. (2020). Self-assembled few-layered MoS_2_ on SnO_2_ anode for enhancing lithium-ion storage. Nanomaterials.

[16] He H., Gan Q., Wang H., Xu G.L., Zhang X., Huang D., Fu F., Tang Y., Amine K., Shao M. (2018). Structure-dependent performance of TiO_2_/C as anode material for Na-ion batteries. Nano Energy.

[17] Ai C., Yin M., Wang C., Sun J. (2004). Synthesis and characterization of spinel type ZnCo_2_O_4_ as a novel anode material for lithium ion batteries. J. Mater. Sci..

[18] Guo H., Zhang Y., Marschilok A.C., Takeuchi K.J., Takeuchi E.S., Liu P. (2017). A first principles study of spinel ZnFe_2_O_4_ for electrode materials in lithium-ion batteries. Phys. Chem. Chem. Phys..

[19] Yao W., Xu Z., Xu X., Xie Y., Qiu W., Xu J., Zhang D. (2018). Two-dimensional holey ZnFe_2_O_4_ nanosheet/reduced graphene oxide hybrids by self-link of nanoparticles for high-rate lithium storage. Electrochim. Acta.

[20] Hsieh C.T., Lin C.Y., Chen Y.F., Lin J.S. (2013). Synthesis of ZnO@Graphene composites as anode materials for lithium ion batteries. Electrochim. Acta.

[21] Lu S., Wang H., Zhou J., Wu X., Qin W. (2017). Atomic layer deposition of ZnO on carbon black as nanostructured anode materials for high-performance lithium-ion batteries. Nanoscale.

[22] Liu N., Lu Z., Zhao J., McDowell M.T., Lee H.W., Zhao W., Cui Y. (2014). A pomegranate-inspired nanoscale design for large-volume-change lithium battery anodes. Nat. Nanotechnol..

[23] Gao H., Zhou W., Jang J.H., Goodenough J.B. (2016). Cross-Linked Chitosan as a Polymer Network Binder for an Antimony Anode in Sodium-Ion Batteries. Adv. Energy Mater..

[24] Li F., Yang L., Xu G., Xiaoqiang H., Yang X., Wei X., Ren Z., Shen G., Han G. (2013). Hydrothermal self-assembly of hierarchical flower-like ZnO nanospheres with nanosheets and their application in Li-ion batteries. J. Alloys Compd..

[25] Park K.T., Xia F., Kim S.W., Kim S.B., Song T., Paik U., Park W.I. (2013). Facile synthesis of ultrathin ZnO nanotubes with well-organized hexagonal nanowalls and sealed layouts: Applications for lithium ion battery anodes. J. Phys. Chem. C.

[26] Huang X.H., Guo R.Q., Wu J.B., Zhang P. (2014). Mesoporous ZnO nanosheets for lithium ion batteries. Mater. Lett..

[27] Zhang G., Hou S., Zhang H., Zeng W., Yan F., Li C.C., Duan H. (2015). High-performance and ultra-stable lithium-ion batteries based on MOF-derived ZnO@ZnO quantum dots/C core-shell nanorod arrays on a carbon cloth anode. Adv. Mater..

[28] Ge X., Li Z., Wang C., Yin L. (2015). Metal-organic frameworks derived porous core/shell structured ZnO/ZnCo_2_O_4_/C hybrids as anodes for high-performance lithium-ion battery. ACS Appl. Mater. Interfaces.

[29] Gan Q., Zhao K., Liu S., He Z. (2017). Solvent-free synthesis of N-doped carbon coated ZnO nanorods composite anode via a ZnO support-induced ZIF-8 in-situ growth strategy. Electrochim. Acta.

[30] Bresser D., Mueller F., Fiedler M., Krueger S., Kloepsch R., Baither D., Winter M., Paillard E., Passerini S. (2013). Transition-metal-doped zinc oxide nanoparticles as a new lithium-ion anode material. Chem. Mater..

[31] Wang X., Zhou X., Yao K., Zhang J., Liu Z. (2011). A SnO_2_/graphene composite as a high stability electrode for lithium ion batteries. Carbon.

[32] Guo W., Sun W., Wang Y. (2015). Multilayer CuO@NiO Hollow Spheres: Microwave-Assisted Metal-Organic-Framework Derivation and Highly Reversible Structure-Matched Stepwise Lithium Storage. ACS Nano.

[33] Belliard F., Irvine J.T.S. (2001). Electrochemical performance of ball-milled ZnO-SnO_2_ systems as anodes in lithium-ion battery. J. Power Sources.

[34] Franken R.H., Van Der Werf C.H.M., Löffler J., Rath J.K., Schropp R.E.I. (2006). Beneficial effects of sputtered ZnO:Al protection layer on SnO_2_:F for high-deposition rate hot-wire CVD p-i-n solar cells. Thin Solid Films.

[35] Wang J., Du N., Zhang H., Yu J., Yang D. (2011). Layer-by-layer assembly synthesis of ZnO/SnO_2_ composite nanowire arrays as high-performance anode for lithium-ion batteries. Mater. Res. Bull..

[36] Ahmad M., Yingying S., Sun H., Shen W., Zhu J. (2012). SnO_2_/ZnO composite structure for the lithium-ion battery electrode. J. Solid State Chem..

[37] Huang Y., Liu X., Lu L., Fang J., Ni H., Ji Z. (2015). Preparation and characterization of ZnO/SnO_2_ composite thin films as high-capacity anode for lithium-ion batteries. Appl. Phys. A.

[38] Xie Q., Ma Y., Zeng D., Wang L., Yue G., Peng D.L. (2014). Facile fabrication of various zinc-nickel citrate microspheres and their transformation to ZnO-NiO hybrid microspheres with excellent lithium storage properties. Sci. Rep..

[39] Pan Q., Zheng F., Ou X., Yang C., Xiong X., Liu M. (2017). MoS_2_ encapsulated SnO_2_-SnS/C nanosheets as a high performance anode material for lithium ion batteries. Chem. Eng. J..

[40] Zhang H., Huang X., Noonan O., Zhou L., Yu C. (2017). Tailored Yolk–Shell Sn@C Nanoboxes for High-Performance Lithium Storage. Adv. Funct. Mater..

[41] Wu Z.S., Ren W., Wen L., Gao L., Zhao J., Chen Z., Zhou G., Li F., Cheng H.M. (2010). Graphene anchored with Co_3_O_4_ nanoparticles as anode of lithium ion batteries with enhanced reversible capacity and cyclic performance. ACS Nano.

[42] Hu Y., Yan C., Chen D., Lv C., Jiao Y., Chen G. (2017). One-dimensional Co_3_O_4_ nanonet with enhanced rate performance for lithium ion batteries: Carbonyl-Β-cyclodextrin inducing and kinetic analysis. Chem. Eng. J..

[43] Yang J., Ouyang Y., Zhang H., Xu H., Zhang Y., Wang Y. (2016). Novel Fe_2_P/graphitized carbon yolk/shell octahedra for high-efficiency hydrogen production and lithium storage. J. Mater. Chem. A.

[44] Yang J., Wu Q., Yang X., He S., Khan J., Meng Y., Zhu X., Tong S., Wu M. (2017). Chestnut-Like TiO_2_@α-Fe_2_O_3_ Core-Shell Nanostructures with Abundant Interfaces for Efficient and Ultralong Life Lithium-Ion Storage. ACS Appl. Mater. Interfaces.

[45] Li J., Yan D., Lu T., Yao Y., Pan L. (2017). An advanced CoSe embedded within porous carbon polyhedra hybrid for high performance lithium-ion and sodium-ion batteries. Chem. Eng. J..

[46] Lin D., Lu Z., Hsu P.C., Lee H.R., Liu N., Zhao J., Wang H., Liu C., Cui Y. (2015). A high tap density secondary silicon particle anode fabricated by scalable mechanical pressing for lithium-ion batteries. Energy Environ. Sci..

[47] Wu C., Maier J., Yu Y. (2015). Sn-Based Nanoparticles Encapsulated in a Porous 3D Graphene Network: Advanced Anodes for High-Rate and Long Life Li-Ion Batteries. Adv. Funct. Mater..

[48] Seng K.H., Li L., Chen D.P., Chen Z.X., Wang X.L., Liu H.K., Guo Z.P. (2013). The effects of FEC (fluoroethylene carbonate) electrolyte additive on the lithium storage properties of NiO (nickel oxide) nanocuboids. Energy.

[49] Wu L., Buchholz D., Bresser D., Gomes Chagas L., Passerini S. (2014). Anatase TiO_2_ nanoparticles for high power sodium-ion anodes. J. Power Sources.

[50] Zou F., Chen Y.M., Liu K., Yu Z., Liang W., Bhaway S.M., Gao M., Zhu Y. (2016). Metal organic frameworks derived hierarchical hollow NiO/Ni/graphene composites for lithium and sodium storage. ACS Nano.

[51] Hu H., Zhang J., Guan B., Lou X.W.D. (2016). Unusual Formation of CoSe@carbon Nanoboxes, which have an Inhomogeneous Shell, for Efficient Lithium Storage. Angew. Chem. Int. Ed..

[52] Sun Y., Hu X., Luo W., Huang Y. (2011). Self-assembled hierarchical MoO_2_/graphene nanoarchitectures and their application as a high-performance anode material for lithium-ion batteries. ACS Nano.

[53] Guo J., Liu Q., Wang C., Zachariah M.R. (2012). Interdispersed amorphous MnO_x_-carbon nanocomposites with superior electrochemical performance as lithium-storage material. Adv. Funct. Mater..

[54] Jamnik J., Maier J. (2003). Nanocrystallinity effects in lithium battery materials: Aspects of nano-ionics. Part IV. Phys. Chem. Chem. Phys..

[55] Xie Q., Li F., Guo H., Wang L., Chen Y., Yue G., Peng D.L. (2013). Template-free synthesis of amorphous double-shelled zinc-cobalt citrate hollow microspheres and their transformation to crystalline ZnCo_2_O_4_ microspheres. ACS Appl. Mater. Interfaces.

[56] Xie Q., Zeng D., Ma Y., Lin L., Wang L., Peng D.L. (2015). Synthesis of ZnO-ZnCo_2_O_4_ hybrid hollow microspheres with excellent lithium storage properties. Electrochim. Acta.

[57] Chao D., Zhu C., Yang P., Xia X., Liu J., Wang J., Fan X., Savilov S.V., Lin J., Fan H.J. (2016). Array of nanosheets render ultrafast and high-capacity Na-ion storage by tunable pseudocapacitance. Nat. Commun..

[58] Chen Z., Wu R., Wang H., Jiang Y., Jin L., Guo Y., Song Y., Fang F., Sun D. (2017). Construction of hybrid hollow architectures by in-situ rooting ultrafine ZnS nanorods within porous carbon polyhedra for enhanced lithium storage properties. Chem. Eng. J..

[59] Li J., Yan D., Hou S., Lu T., Yao Y., Chua D.H.C., Pan L. (2018). Metal-organic frameworks derived yolk-shell ZnO/NiO microspheres as high-performance anode materials for lithium-ion batteries. Chem. Eng. J..

[60] Wang C., Zhao Y., Zhou L., Liu Y., Zhang W., Zhao Z., Hozzein W.N., Alharbi H.M.S., Li W., Zhao D. (2018). Mesoporous carbon matrix confinement synthesis of ultrasmall WO_3_ nanocrystals for lithium ion batteries. J. Mater. Chem. A.

[61] Wu X., Yao S. (2017). Flexible electrode materials based on WO_3_ nanotube bundles for high performance energy storage devices. Nano Energy.

[62] Tu C., Zhang Z., Shao A., Qi X., Zhu C., Li C., Yang Z. (2021). Constructing a directional ion acceleration layer at WO_3_/ZnO heterointerface to enhance Li-ion transfer and storage. Compos. Part B Eng..

[63] Su D., Kim H.S., Kim W.S., Wang G. (2012). Synthesis of tuneable porous hematites (α-Fe_2_O_3_) for gas sensing and lithium storage in lithium ion batteries. Microporous Mesoporous Mater..

[64] Karunakaran G., Kundu M., Kumari S., Kolesnikov E., Gorshenkov M.V., Maduraiveeran G., Sasidharan M., Kuznetsov D. (2018). ZnO/Cu_2_MgO_3_ hollow porous nanocage: A new class of hybrid anode material for advanced lithium-ion batteries. J. Alloys Compd..

[65] Li Z., Li B., Yin L., Qi Y. (2014). Prussion blue-supported annealing chemical reaction route synthesized double-shelled Fe_2_O_3_/Co_3_O_4_ hollow microcubes as anode materials for Lithium-Ion battery. ACS Appl. Mater. Interfaces.

[66] Zhang L., Wu H.B., Madhavi S., Hng H.H., Lou X.W. (2012). Formation of Fe_2_O_3_ microboxes with hierarchical shell structures from metal-organic frameworks and their lithium storage properties. J. Am. Chem. Soc..

[67] Yan N., Hu L., Li Y., Wang Y., Zhong H., Hu X., Kong X., Chen Q. (2012). Co_3_O_4_ nanocages for high-performance anode material in lithium-ion batteries. J. Phys. Chem. C.

[68] Nie P., Shen L., Luo H., Ding B., Xu G., Wang J., Zhang X. (2014). Prussian blue analogues: A new class of anode materials for lithium ion batteries. J. Mater. Chem. A.

[69] Huang G., Zhang F., Zhang L., Du X., Wang J., Wang L. (2014). Hierarchical NiFe_2_O_4_/Fe_2_O_3_ nanotubes derived from metal organic frameworks for superior lithium ion battery anodes. J. Mater. Chem. A.

[70] Zou L., Li F., Xiang X., Evans D.G., Duan X. (2006). Self-generated template pathway to high-surface-area zinc aluminate spinel with mesopore network from a single-source inorganic precursor. Chem. Mater..

[71] Hou L., Lian L., Zhang L., Pang G., Yuan C., Zhang X. (2015). Self-sacrifice template fabrication of hierarchical mesoporous bi-component-active ZnO/ZnFe_2_O_4_ sub-microcubes as superior anode towards high-performance lithium-ion battery. Adv. Funct. Mater..

[72] Yang S.J., Nam S., Kim T., Im J.H., Jung H., Kang J.H., Wi S., Park B., Park C.R. (2013). Preparation and exceptional lithium anodic performance of porous carbon-coated ZnO quantum dots derived from a metal-organic framework. J. Am. Chem. Soc..

[73] Zou F., Hu X., Li Z., Qie L., Hu C., Zeng R., Jiang Y., Huang Y. (2014). MOF-derived porous ZnO/ZnFe_2_O_4_/C octahedra with hollow interiors for high-rate lithium-ion batteries. Adv. Mater..

[74] Bresser D., Paillard E., Kloepsch R., Krueger S., Fiedler M., Schmitz R., Baither D., Winter M., Passerini S. (2013). Carbon coated ZnFe_2_O_4_ nanoparticles for advanced lithium-ion anodes. Adv. Energy Mater..

[75] Zheng F., He M., Yang Y., Chen Q. (2015). Nano electrochemical reactors of Fe_2_O_3_ nanoparticles embedded in shells of nitrogen-doped hollow carbon spheres as high-performance anodes for lithium-ion batteries. Nanoscale.

[76] Wu Z.S., Ren W., Xu L., Li F., Cheng H.M. (2011). Doped graphene sheets as anode materials with superhigh rate and large capacity for lithium ion batteries. ACS Nano.

[77] Zhao Y., Huang G., Li Y., Edy R., Gao P., Tang H., Bao Z., Mei Y. (2018). Three-dimensional carbon/ZnO nanomembrane foam as an anode for lithium-ion battery with long-life and high areal capacity. J. Mater. Chem. A.

[78] Hsieh C.T., Lin J.Y., Mo C.Y. (2011). Improved storage capacity and rate capability of Fe_3_O_4_-graphene anodes for lithium-ion batteries. Electrochim. Acta.

[79] Zhang M., Lei D., Yin X., Chen L., Li Q., Wang Y., Wang T. (2010). Magnetite/graphene composites: Microwave irradiation synthesis and enhanced cycling and rate performances for lithium ion batteries. J. Mater. Chem..

[80] Yu Z., Zhu S., Li Y., Liu Q., Feng C., Zhang D. (2011). Synthesis of SnO_2_ nanoparticles inside mesoporous carbon via a sonochemical method for highly reversible lithium batteries. Mater. Lett..

[81] Liu J., Zhou Y., Liu F., Liu C., Wang J., Pan Y., Xue D. (2012). One-pot synthesis of mesoporous interconnected carbon-encapsulated Fe_3_O_4_ nanospheres as superior anodes for Li-ion batteries. RSC Adv..

[82] Liu B., Wang X., Liu B., Wang Q., Tan D., Song W., Hou X., Chen D., Shen G. (2013). Advanced rechargeable lithium-ion batteries based on bendable ZnCo_2_O_4_-urchins-on-carbon-fibers electrodes. Nano Res..

[83] Han Q., Li X., Wang F., Han Z., Geng D., Zhang W., Li Y., Deng Y., Zhang J., Niu S. (2019). Carbon fiber@ pore-ZnO composite as anode materials for structural lithium-ion batteries. J. Electroanal. Chem..

[84] Nardecchia S., Carriazo D., Ferrer M.L., Gutiérrez M.C., Monte F.D. (2013). Three dimensional macroporous architectures and aerogels built of carbon nanotubes and/or graphene: Synthesis and applications. Chem. Soc. Rev..

[85] Son D.I., Kwon B.W., Park D.H., Seo W.S., Yi Y., Angadi B., Lee C.L., Choi W.K. (2012). Emissive ZnO-graphene quantum dots for white-light-emitting diodes. Nat. Nanotechnol..

[86] Dou Y., Xu J., Ruan B., Liu Q., Pan Y., Sun Z., Dou S.X. (2016). Atomic Layer-by-Layer Co_3_O_4_/Graphene Composite for High Performance Lithium-Ion Batteries. Adv. Energy Mater..

[87] Fan L., Zhang Y., Zhang Q., Wu X., Cheng J., Zhang N., Feng Y., Sun K. (2016). Graphene Aerogels with Anchored Sub-Micrometer Mulberry-Like ZnO Particles for High-Rate and Long-Cycle Anode Materials in Lithium Ion Batteries. Small.

[88] Zhang Y., Wei Y., Li H., Zhao Y., Yin F., Wang X. (2016). Simple fabrication of free-standing ZnO/graphene/carbon nanotube composite anode for lithium-ion batteries. Mater. Lett..

[89] Sun H., Mei L., Liang J., Zhao Z., Lee C., Fei H., Ding M., Lau J., Li M., Wang C. (2017). Three-dimensional holey-graphene/niobia composite architectures for ultrahigh-rate energy storage. Science.

[90] Lv D., Zheng J., Li Q., Xie X., Ferrara S., Nie Z., Mehdi L.B., Browning N.D., Zhang J.G., Graff G.L. (2015). High Energy Density Lithium-Sulfur Batteries: Challenges of Thick Sulfur Cathodes. Adv. Energy Mater..

[91] Gallagher K.G., Trask S.E., Bauer C., Woehrle T., Lux S.F., Tschech M., Lamp P., Polzin B.J., Ha S., Long B. (2016). Optimizing Areal Capacities through Understanding the Limitations of Lithium-Ion Electrodes. J. Electrochem. Soc..

[92] Peng H.J., Huang J.Q., Cheng X.B., Zhang Q. (2017). Review on High-Loading and High-Energy Lithium–Sulfur Batteries. Adv. Energy Mater..

[93] Liu J., Liu X.W. (2012). Two-dimensional nanoarchitectures for lithium storage. Adv. Mater..

[94] Si W., Mönch I., Yan C., Deng J., Li S., Lin G., Han L., Mei Y., Schmidt O.G. (2014). A single rolled-up Si tube battery for the study of electrochemical kinetics, electrical conductivity, and structural integrity. Adv. Mater..

[95] Zhou G., Li L., Ma C., Wang S., Shi Y., Koratkar N., Ren W., Li F., Cheng H.M. (2015). A graphene foam electrode with high sulfur loading for flexible and high energy Li-S batteries. Nano Energy.

[96] Fang R., Zhao S., Hou P., Cheng M., Wang S., Cheng H.M., Liu C., Li F. (2016). 3D Interconnected Electrode Materials with Ultrahigh Areal Sulfur Loading for Li-S Batteries. Adv. Mater..

[97] Afanasov I.M., Lebedev O.I., Kolozhvary B.A., Smirnov A.V., van Tendeloo G. (2011). Nickel/Carbon composite materials based on expanded graphite. New Carbon Mater..

[98] Wang L., Zhu Y., Guo C., Zhu X., Liang J., Qian Y. (2014). Ferric chloride-graphite intercalation compounds as anode materials for Li-ion batteries. ChemSusChem.

[99] Ma C., Ma C., Wang J., Wang H., Shi J., Song Y., Guo Q., Liu L. (2014). Exfoliated graphite as a flexible and conductive support for Si-based Li-ion battery anodes. Carbon.

[100] Jiang B., Tian C., Zhou W., Wang J., Xie Y., Pan Q., Ren Z., Dong Y., Fu D., Han J. (2011). In situ growth of TiO_2_ in interlayers of expanded graphite for the fabrication of TiO_2_-graphene with enhanced photocatalytic activity. Chem. Eur. J..

[101] Zhang W., Wan W., Zhou H., Chen J., Wang X., Zhang X. (2013). In-situ synthesis of magnetite/expanded graphite composite material as high rate negative electrode for rechargeable lithium batteries. J. Power Sources.

[102] Naderi H.R., Mortaheb H.R., Zolfaghari A. (2014). Supercapacitive properties of nanostructured MnO_2_/exfoliated graphite synthesized by ultrasonic vibration. J. Electroanal. Chem..

[103] Huang Y.G., Lin X.L., Zhang X.H., Pan Q.C., Yan Z.X., Wang H.Q., Chen J.J., Li Q.Y. (2015). Fe_3_C@carbon nanocapsules/expanded graphite as anode materials for lithium ion batteries. Electrochim. Acta.

[104] Huang Y., Lin X., Pan Q., Li Q., Zhang X., Yan Z., Wu X., He Z., Wang H. (2016). Al@C/Expanded Graphite Composite as Anode Material for Lithium Ion Batteries. Electrochim. Acta.

[105] Hu S., Song Y., Yuan S., Liu H., Xu Q., Wang Y., Wang C.X., Xia Y.Y. (2016). A hierarchical structure of carbon-coated Li_3_VO_4_ nanoparticles embedded in expanded graphite for high performance lithium ion battery. J. Power Sources.

[106] Li Y., Zhao Y., Huang G., Xu B., Wang B., Pan R., Men C., Mei Y. (2017). ZnO Nanomembrane/Expanded Graphite Composite Synthesized by Atomic Layer Deposition as Binder-Free Anode for Lithium Ion Batteries. ACS Appl. Mater. Interfaces.

[107] Wu S., Wang W., Li M., Cao L., Lyu F., Yang M., Wang Z., Shi Y., Nan B., Yu S. (2016). Highly durable organic electrode for sodium-ion batteries via a stabilized α-C radical intermediate. Nat. Commun..

[108] Wu S., Zhu Y., Huo Y., Luo Y., Zhang L., Wan Y., Nan B., Cao L., Wang Z., Li M. (2017). Bimetallic organic frameworks derived CuNi/carbon nanocomposites as efficient electrocatalysts for oxygen reduction reaction. Sci. China Mater..

[109] Duan Z.Q., Liu Y.T., Xie X.M., Ye X.Y., Zhu X.D. (2016). H-BN Nanosheets as 2D Substrates to Load 0D Fe_3_O_4_ Nanoparticles: A Hybrid Anode Material for Lithium-Ion Batteries. Chem. Asian J..

[110] Quartarone E., Dall’asta V., Resmini A., Tealdi C., Tredici I.G., Tamburini U.A., Mustarelli P. (2016). Graphite-coated ZnO nanosheets as high-capacity, highly stable, and binder-free anodes for lithium-ion batteries. J. Power Sources.

[111] Li Z., Yin L. (2015). Sandwich-like reduced graphene oxide wrapped MOF-derived ZnCo_2_O_4_-ZnO-C on nickel foam as anodes for high performance lithium ion batteries. J. Mater. Chem. A.

[112] Gan Q., Liu S., Zhao K., Wu Y., He Z., Zhou Z. (2016). Graphene supported nitrogen-doped porous carbon nanosheets derived from zeolitic imidazolate framework for high performance supercapacitors. RSC Adv..

[113] Li C., Hu Q., Li Y., Zhou H., Lv Z., Yang X., Liu L., Guo H. (2016). Hierarchical hollow Fe_2_O_3_ @MIL-101(Fe)/C derived from metal-organic frameworks for superior sodium storage. Sci. Rep..

[114] Yu L., Liu J., Xu X., Zhang L., Hu R., Liu J., Yang L., Zhu M. (2017). Metal-organic framework-derived NiSb alloy embedded in carbon hollow spheres as superior lithium-ion battery anodes. ACS Appl. Mater. Interfaces.

[115] Thauer E., Zakharova G.S., Andreikov E.I., Adam V., Wegener S.A., Nölke J.H., Singer L., Ottmann A., Asyuda A., Zharnikov M. (2021). Novel synthesis and electrochemical investigations of ZnO/C composites for lithium-ion batteries. J. Mater. Sci..

[116] Eisenmann T., Birrozzi A., Mullaliu A., Giuli G., Trapananti A., Passerini S., Bresser D. (2021). Effect of Applying a Carbon Coating on the Crystal Structure and De-/Lithiation Mechanism of Mn-Doped ZnO Lithium-Ion Anodes. J. Electrochem. Soc..

[117] Köse H., Dombaycıoğlu Ş., Aydın A.O., Akbulut H. (2016). Production and characterization of free-standing ZnO/SnO_2_/MWCNT ternary nanocomposite Li-ion battery anode. Int. J. Hydrog. Energy.

[118] Zhang J., Zhu Y., Cao C., Butt F.K. (2015). Microwave-assisted and large-scale synthesis of SnO_2_/carbon-nanotube hybrids with high lithium storage capacity. RSC Adv..

[119] Guler M.O., Cetinkaya T., Tocoglu U., Akbulut H. (2014). Electrochemical performance of MWCNT reinforced ZnO anodes for Li-ion batteries. Microelectron. Eng..

[120] Kuwabata S., Masui S., Yoneyama H. (1999). Charge–discharge properties of composites of LiMn_2_O_4_ and polypyrrole as positive electrode materials for 4 V class of rechargeable Li batteries. Electrochim. Acta.

[121] Tang W., Liu L., Zhu Y., Sun H., Wu Y., Zhu K. (2012). An aqueous rechargeable lithium battery of excellent rate capability based on a nanocomposite of MoO_3_ coated with PPy and LiMn_2_O_4_. Energy Environ. Sci..

[122] Zhao J., Zhang S., Liu W., Du Z., Fang H. (2014). Fe_3_O_4_/PPy composite nanospheres as anode for lithium-ion batteries with superior cycling performance. Electrochim. Acta.

[123] Zhong X.B., Wang H.Y., Yang Z.Z., Jin B., Jiang Q.C. (2015). Facile synthesis of mesoporous ZnCo_2_O_4_ coated with polypyrrole as an anode material for lithium-ion batteries. J. Power Sources.

[124] Zhang J., Li L., Chen J., He N., Yu K., Liang C. (2021). Controllable SnO_2_/ZnO@PPy hollow nanotubes prepared by electrospinning technology used as anode for lithium ion battery. J. Phys. Chem. Solids.

[125] Köse H., Aydin A.O., Akbulut H. (2014). Free-standing SnO_2_/MWCNT nanocomposite anodes produced by different rate spin coatings for Li-ion batteries. Int. J. Hydrog. Energy.

[126] Zhao Y., Li X., Dong L., Yan B., Shan H., Li D., Sun X. (2015). Electrospun SnO_2_-ZnO nanofibers with improved electrochemical performance as anode materials for lithium-ion batteries. Int. J. Hydrog. Energy.

[127] Joshi B.N., An S., Jo H.S., Song K.Y., Park H.G., Hwang S., Al-Deyab S.S., Yoon W.Y., Yoon S.S. (2016). Flexible, Freestanding, and Binder-free SnO_x_-ZnO/Carbon Nanofiber Composites for Lithium Ion Battery Anodes. ACS Appl. Mater. Interfaces.

[128] Qiao L., Wang X., Sun X., Li X., Zheng Y., He D. (2013). Single electrospun porous NiO-ZnO hybrid nanofibers as anode materials for advanced lithium-ion batteries. Nanoscale.

[129] Ma L., Pei X.Y., Mo D.C., Lyu S.S., Fu Y.X. (2018). Fabrication of NiO-ZnO/RGO composite as an anode material for lithium-ion batteries. Ceram. Int..

[130] Wang X.L., Han W.Q., Chen H., Bai J., Tyson T.A., Yu X.Q., Wang X.J., Yang X.Q. (2011). Amorphous hierarchical porous GeO_x_ as high-capacity anodes for Li ion batteries with very long cycling life. J. Am. Chem. Soc..

[131] Jin S., Li N., Cui H., Wang C. (2013). Growth of the vertically aligned graphene@ amorphous GeO_x_ sandwich nanoflakes and excellent Li storage properties. Nano Energy.

[132] Medvedev A.G., Mikhaylov A.A., Grishanov D.A., Yu D.Y.W., Gun J., Sladkevich S., Lev O., Prikhodchenko P.V. (2017). GeO_2_ Thin Film Deposition on Graphene Oxide by the Hydrogen Peroxide Route: Evaluation for Lithium-Ion Battery Anode. ACS Appl. Mater. Interfaces.

[133] He X., Hu Y., Chen R., Shen Z., Wu K., Cheng Z., Pan P. (2019). Foldable uniform GeO_x_/ZnO/C composite nanofibers as a high-capacity anode material for flexible lithium ion batteries. Chem. Eng. J..

[134] Zhang Y.Z., Wang Y., Xie Y.L., Cheng T., Lai W.Y., Pang H., Huang W. (2014). Porous hollow Co_3_O_4_ with rhombic dodecahedral structures for high-performance supercapacitors. Nanoscale.

[135] Liu B., Zhang J., Wang X., Chen G., Chen D., Zhou C., Shen G. (2012). Hierarchical three-dimensional ZnCo_2_O_4_ nanowire arrays/carbon cloth anodes for a novel class of high-performance flexible lithium-ion batteries. Nano Lett..

[136] Du N., Xu Y., Zhang H., Yu J., Zhai C., Yang D. (2011). Porous ZnCo_2_O_4_ nanowires synthesis via sacrificial templates: High-performance anode materials of li-ion batteries. Inorg. Chem..

[137] Luo W., Hu X., Sun Y., Huang Y. (2012). Electrospun porous ZnCo_2_O_4_ nanotubes as a high-performance anode material for lithium-ion batteries. J. Mater. Chem..

[138] Giri A.K., Pal P., Ananthakumar R., Jayachandran M., Mahanty S., Panda A.B. (2014). 3D hierarchically assembled porous wrinkled-paper-like structure of ZnCo_2_O_4_ and Co-ZnO@C as anode materials for lithium-ion batteries. Cryst. Growth Des..

[139] Zhong J., Cao C., Liu Y., Li Y., Khan W.S. (2010). Hollow core-shell η-Fe_2_O_3_ microspheres with excellent lithium-storage and gas-sensing properties. Chem. Commun..

[140] Zhou L., Xu H., Zhang H., Yang J., Hartono S.B., Qian K., Zou J., Yu C. (2013). Cheap and scalable synthesis of α-Fe_2_O_3_ multi-shelled hollow spheres as high-performance anode materials for lithium ion batteries. Chem. Commun..

[141] Lin H.B., Rong H.B., Huang W.Z., Liao Y.H., Xing L.D., Xu M.Q., Li X.P., Li W.S. (2014). Triple-shelled Mn_2_O_3_ hollow nanocubes: Force-induced synthesis and excellent performance as the anode in lithium-ion batteries. J. Mater. Chem. A.

[142] Ma Y., Ma Y., Geiger D., Kaiser U., Zhang H., Kim G.T., Diemant T., Behm R.J., Varzi A., Passerini S. (2017). ZnO/ZnFe_2_O_4_/N-doped C micro-polyhedrons with hierarchical hollow structure as high-performance anodes for lithium-ion batteries. Nano Energy.

